# Environmental and Host Blood Interactions Shape *Yersinia pestis* Dynamics in the Rat Flea, *Xenopsylla cheopis*

**DOI:** 10.3390/pathogens15060639

**Published:** 2026-06-16

**Authors:** Cassandra D. Pauling, Deborah M. Anderson

**Affiliations:** Department of Pathobiology and Integrative Biomedical Sciences, University of Missouri, Columbia, MO 65211, USA; pauling@ucmo.edu

**Keywords:** *Yersinia pestis*, *Xenopsylla cheopis*, flea, plague, transmission

## Abstract

*Yersinia pestis* is the causative agent of bubonic plague, a zoonotic disease that is primarily transmitted by infectious fleas. Plague is endemic in regions around the world, including the United States, where optimal climate conditions support a stable, enzootic sylvatic transmission cycle. Epizootic outbreaks periodically occur with rapid spread of disease that increases the risk of human exposure. As fleas are ectotherms that are responsive to environmental conditions, it is likely that transmission efficiency varies under different ecological conditions, with optimal conditions capable of supporting rapid spread of disease while sub-optimal conditions may promote lower levels of transmission. To test this, we experimentally infected *Xenopsylla cheopis* with *Y. pestis* using a membrane feeder in order to define the impact of varying temperature, humidity and mammalian blood sources on infection and transmission. We show that environmental factors and host blood source are key factors influencing colonization, bacterial aggregation, and transmission rates, with variation in the responses seen depending on the experimental conditions. The combined data illustrate the impact of ecological factors on *Y. pestis* flea infection and suggests that optimal conditions involving the vector–host–pathogen interface are needed for enhanced transmission rates and the rapid spread of infection that occurs during epizootic outbreaks.

## 1. Introduction

Bubonic plague, caused by the Gram-negative bacterium *Yersinia pestis*, begins when an infected flea transmits the pathogen to a susceptible animal host, leading to infection of the nearest lymph node [1,2,3]. From there, the infection disseminates and bacteria grow in secondary and tertiary immune tissues as well as the liver, lungs and blood. Treatment with antibiotics can stop the infection, especially in the early stage prior to systemic spread of *Y. pestis* [4]. However, the disease is challenging for medical treatment as toxic inflammatory responses quickly consume the patient. Because of the challenges of treatment of plague, understanding the zoonotic transmission cycle is a high-priority research area for reducing the risk of human exposure to *Y. pestis*-infected fleas [5].

The persistence of plague in the environment occurs around the world, yet the infection of the vertebrate and invertebrate hosts often results in a high degree of mortality [6]. With both hosts developing a lethal infection, it is difficult to understand how an enzootic cycle might persist in nature. Strong genetic and microbiological data indicate that *Y. pestis* biofilm is essential for flea transmission, yet fleas with complete proventricular blockage are unable to feed in spite of repeated attempts [7,8]. While this may enhance transmission rates by causing the flea to regurgitate and feed repeatedly, paradoxically, it also causes the flea to become dehydrated and accelerates its mortality [9,10,11,12]. Proventricular blockage is highly variable across flea species, with the rat flea, *Xenopsylla cheopis*, exhibiting considerably higher rates of blockage than other species. In addition to biofilm-dependent transmission, many flea species that vector plague to humans and animals appear capable of transmitting *Y. pestis* shortly after pathogen acquisition, a process termed early-phase transmission (EPT) [13,14,15,16,17,18]. This transmission mechanism is thought to be independent of biofilm [19]. The efficiency rate of EPT appears variable and is positively influenced by higher numbers of infected fleas feeding on the mammalian host [12,13,20]. As increased flea burdens on individual animals have been documented during epizootic episodes of plague, there may be significant contributions made by EPT to rapid spread of disease [21]. Conversely, low-level EPT transmission events may result in less severe disease outcomes that may not support epizootic spread [20]. Little is known about the underlying mechanism of EPT, and no bacterial genes have yet been identified as essential for this transmission process. Nevertheless, many flea species have demonstrated the capacity for EPT under laboratory conditions [14,15,16,18]. Recent data suggest high bacterial load in the esophagus, often present in the early stage of infection, may be sufficient to trigger regurgitative transmission [22]. Thus, it may be that EPT and biofilm-dependent transmission are both regurgitative mechanisms that may be enhanced by bacterial aggregation in the proventriculus of the flea host.

The sylvatic plague cycle occurs in many geographic regions, where it alternates between enzootic or quiescent phases and epizootic outbreaks. Animal and human cases of disease are nearly always seasonal, even in areas with mild winters, and are associated with the seasonality of fleas [23,24,25,26,27]. While flea and rodent abundance is clearly increased by warm climate, there is also strong evidence for optimal conditions of temperature and precipitation that support epizootic outbreaks, suggesting relative humidity may also be important to plague ecology [28,29,30,31,32]. For example, increased annual precipitation often precedes high epizootic plague activity in the following year, although drought has been reported to be associated with increased incidence of plague in prairie dogs, leaving the importance of relative humidity unclear [32]. Soil chemistry is also thought to be associated with the plague cycle, with optimal salinity, trace minerals and moisture capacity supporting increased flea transmission of plague [33,34,35,36,37,38]. These environmental factors directly influence flea physiology and therefore also the host–pathogen interactions that facilitate the transmission cycle.

Host species’ blood varies in lipid and protein composition, and previous work has established that oviposition and reproduction of fleas vary with different host species’ blood [39]. Likewise, *Y. pestis* gene expression is known to be influenced by temperature, with multiple mechanisms in place that ensure optimal bacterial growth in the flea and mammalian hosts. Multiple lines of evidence indicate that *Y. pestis* interactions in the blood contribute significantly to the establishment of a transmissible infection in the flea host [22,40]. For example, rat blood has been shown to contain oxyhemoglobin crystals, which are associated with the development of esophageal blockage that may enhance regurgitative transmission, a process termed post-infectious esophageal reflux (PIER). The contribution of PIER to the transmission cycle is not yet known.

Digestion of a blood meal by fleas begins with laceration of red blood cells in the proventriculus [41]. Within hours after ingestion, expression of trypsin/chymotrypsin family proteases is increased, initiating protein degradation and generating peptides. Further activation of chymotrypsin expression in the midgut further breaks down these peptides into amino acids used for oogenesis. The digestive process occurs over approximately 12–24 h, during which the flea midgut environment is enriched with proteases. Bacteria ingested with the blood meal induce an immune response during the first 1–3 days after infection, characterized by the production of antimicrobial peptides in the midgut [42,43]. The kinetics of these digestive and immune processes suggest that blood meal processing may influence the efficiency of EPT.

As an ectotherm, the physiological processes of fleas, including respiration, reproduction, and digestion, are sensitive to changes in abiotic factors [41,44,45]. Given the likelihood that respiration, reproduction and successful *Y. pestis* infection are affected by the physiological process of blood meal digestion, it is likely that complex inter-dependencies occur that ultimately drive the seasonality of plague and define optimal conditions for epizootic outbreaks. In this work, we investigated this hypothesis in the laboratory by controlling environmental conditions of temperature and humidity during transmission to and from fleas using an artificial membrane feeder as a model system.

## 2. Materials and Methods

Bacteria and strains. This work utilized a laboratory strain of *Yersinia pestis* KIM6+, which lacks the type III secretion system plasmid pCD1 but carries the other two native plasmids, pMT1 and pPCP1 [46,47,48]. This strain also carries pNE160, which encodes the fluorescent protein tdTomato (excitation: 554 nm; emission: 581 nm) constitutively expressed from the *cysZ* promoter [49]. The recombinant derivative of KIM6+ used in this work did not reintroduce the pCD1 plasmid and, therefore, is classified as a select agent-exempt strain by the US Centers for Disease Control and Prevention. All usage of *Y. pestis* KIM6+ was approved by the University of Missouri Institutional Biosafety Committee prior to initiating the study. For infections, *Y. pestis* KIM6+pNE160 was routinely streaked from frozen stock and grown on heart infusion agar (HIA) with 100 µg/mL ampicillin for 48–96 h at 26 °C. Liquid cultures were prepared by inoculating a single colony into HIB with ampicillin, then growing for 18–24 h at 26 °C, 120 rpm shaking. Flea samples were plated on Yersinia selective agar (YSA) to reduce potential contamination by resident microbes.

Flea rearing. Fleas (*Xenopsylla cheopis*) were originally obtained from the NIH/NIAID Rocky Mountain Laboratory, Hamilton, Montana, USA, and were reared at the University of Missouri as previously described [50,51]. Briefly, fleas were housed in a medium composed of food mix (containing equal parts powdered bovine blood, dry milk, and ground mouse chow) and sawdust (1 part food mix: 3 parts sawdust) under conditions of 25 °C, 70% humidity and a 24 h dark cycle; colony feeding was conducted 2 times per week.

Animals. Adult male and female C57BL/6J mice (*Mus musculus*) were originally purchased from the Jackson Laboratory (Maine, USA), then were bred and reared at the University of Missouri under conditions of a 12 h light/12 h dark cycle, 21–23 °C, and 30–70% humidity, with mouse chow and water provided ad libitum. Male and female neonatal mice (less than 6 days old) were used for colony maintenance blood feeding; adult male or female mice (>12 weeks old) were used as the source for the artificial membrane feeder as well as a blood source for experiments where indicated. Other blood sources for infection in the artificial feeder were as follows: rat (*Rattus norvegicus*, BioChemed Services, Winchester, VT, USA), prairie dog (*Cynomys ludovicianus*, PMS Recycled Vermin, Lubbock, TX, USA), and pig (*Sus scrofa* (Ossabaw swine), Corvus Biomedical, Crawfordsville, IN, USA), all collected in sodium heparin tubes. After infection in the indicated host species’ blood, fleas were provided with a blood meal every 7 days using the same host blood source through the membrane feeder. All procedures involving animals were approved prior to initiating the study by the University of Missouri Animal Care and Use Committee.

Flea infections. Prior to use in infection, laboratory-reared, naive *X. cheopis* were separated from the colony in groups of approximately 50 fleas. The fleas were starved for 5–7 days then fed an inoculated blood meal, containing between 5 × 10^8^ and 1 × 10^9^ *Y. pestis*/mL, through an artificial membrane feeder as previously described [50,51]. Briefly, skin from an adult mouse was mounted to the bottom of the glass feeder, and blood containing *Y. pestis* was introduced into the feeder, contacting the skin. A container holding fleas was then placed on the bottom of the feeder, on the adjacent side of the skin, and secured. The feeder was attached to a 37 °C water bath to maintain a blood meal temperature representative of the mammalian host. Fleas were permitted to feed for one hour, then removed and observed under the stereomicroscope for blood meal intake. Fleas that did not feed were removed from the study. Infected fleas were housed in 15 mL conical tubes containing sawdust until processing. The tubes were covered with a fine mesh and maintained upright at the indicated temperature and relative humidity.

Infection assay. Fleas were euthanized at the indicated time points by placing them into a −20 °C freezer for at least 1 h. For each data point, three fleas were removed, rinsed 3 times in sterile ddH_2_O, and the midguts were dissected and placed in 30 μL of sterile phosphate buffered saline (PBS), with vigorous pipetting to homogenize the tissue. For each data point, there were 3 midguts that were pooled and then serially diluted in PBS and plated on YSA.

Chymotrypsin-like serine protease gene expression. Groups of 3 fleas per time point were collected and placed at −80 °C until processing within one week of collection. The fleas were washed in ddH_2_O and placed in sterile RNAse-free PBS on a slide, where the midguts were dissected under a microscope; then, each midgut sample was transferred to 10 µL of PBS. The contents were then placed into an RNAse-free 1.5 mL tube with 200 µL of TRIzol reagent and stored at −80 °C until further processing. mRNA was isolated using the Direct-Zol^TM^ RNA miniprep kit (Zymo Research, Irvine, CA, USA). cDNA was synthesized from 1 ng of isolated RNA using MMLV reverse transcriptase (Promega, Madison, WI, USA). Primers targeting the previously described chymotrypsin-like serine protease (*CfSP*) identified in *Ctenocephalides felis* (GenBank: AF053912.1) were designed using Primer3 [52,53]. Power SYBR green PCR master mix (Fisherbrand^TM^ SYBR^TM^) and gene-specific primers (Table 1) were used in a 20 µL volume reaction with the following conditions: 50 °C for 2 min, 95 °C for 10 min, 40 cycles of 95 °C for 15 s, and 60 °C for 1 min. Reactions and downstream analysis were carried out on the QuantStudioTM 3 system and software (ThermoFisher Scientific, Waltham, MA, USA). Elongation factor 1 delta (ef-1d) was used to normalize the samples [54,55]. Based on amplification plots, the lower threshold for positive was 36 cycles.

Analysis of digestion rates. Protein levels: Total protein levels in fleas were quantified at 6, 12 and 18 h post-feeding to measure peak digestion and digestive decline. Fleas were euthanized at the indicated time points by placing them in a −80 °C freezer for at least 1 h. For protein analysis, the BCA Protein Assay Kit (Pierce Biotechnology, Waltham, MA, USA) was used. For each time point, 3 fleas were washed in sterile PBS, and each midgut was dissected in 10 µL of sterile PBS for a total of 30 µL per time point. Samples were analyzed in triplicate according to the manufacturer’s protocol and quantified using a standard curve of serial dilution of bovine serum albumin (BSA).

Confocal microscopy.Midguts were imaged using a Leica SP8 confocal microscope (Leica, Deerfield, IL, USA) with settings of 1024 × 1024 pixels, a numerical aperture of 0.4, a refractive index of 1, a pinhole of 1 AU, an excitation of 554 nm, and an emission of 580 nm. Image J version 1.54 or Fiji version 2.9 software was used to visualize localization of bacteria and to quantify the fluorescent signal [56,57]. Quantification of proventricular colonization by microscopy occurred as previously described [51]. Briefly, images were converted to 8-bit grayscale and the fluorescence quantified as mean gray value. Relative fluorescent units (RFUs) were calculated as the product of the area of the sample and the mean gray value. Control fleas (n = 10) were given blood that was not infected and analyzed 1 day later to calculate autofluorescence/background signal. Background RFU signal was subtracted from the sample RFU values. Proventricular colonization was defined as measurable bacterial tdTomato fluorescence on the proventricular spines; PIER was defined as esophageal localization of fluorescence.

Mass transmission assay. Groups of approximately 80 fleas were divided into 8 groups of 10, with two groups maintained under each environmental condition as indicated. One group was analyzed for transmission on day 3 and one on day 7. For the transmission assay, groups of 10 fleas were fed on an artificial membrane feeder apparatus with sterile blood, species-matched to the initial infection. Feeding occurred for one hour. Blood from the feeding procedure was collected, serially diluted, and plated to enumerate transmitted *Y. pestis*. The skin membrane was washed three times, with each wash collected for plating; then, the skin was homogenized and plated. All samples were plated on YSA and grown at 26 °C for 4–7 days.

*Statistical analysis:* All data from all trials were evaluated for statistical significance using SPSS v27 (IBM, New York, NY, USA) according to the methods detailed below. Significance was concluded when *p* < 0.05.

For protein and gene expression data obtained during blood meal digestion, data were collected from three independent biological replicates for each blood meal and environmental condition. Protein digestion values were continuous, positive, and right-skewed; therefore, digestion data were analyzed using generalized linear models (GLMs) with a Gamma distribution and a log link function. General and host-specific effects were analyzed using an all-species model, which included host, environmental condition, infection status, and their two-way and three-way interactions. Species-specific models included environmental condition, infection status, and their interaction. Pairwise comparisons were adjusted using Bonferroni correction when applicable.

For CFU data, some samples had undetectable bacteria which were assigned values 0 < X < 1 prior to analysis. CFU data were not normally distributed and were analyzed using a nonparametric Kruskal–Wallis H test followed by Dunn’s post hoc comparisons with Bonferroni correction. Data from all trials were pooled for analysis.

RFU datasets included biologically meaningful zero and near-zero values representing minimal bacterial burden. These data were analyzed by day post-infection using GLMs with a Tweedie distribution and log link function, selected to accommodate zero/near-zero values alongside positive, continuous, right-skewed observations. Analyses included four hosts and four environmental conditions across four time points for a total of 64 sets, with most groups containing ten biological replicates (57 groups with n = 10, 5 with n = 9, 1 with n = 8, and 1 with n = 7). Two complementary analyses were used to evaluate both general and host-specific effects. The all-species model included host, environment, and their interaction, whereas the host-specific models evaluated environmental effects independently. Model fit was assessed using Akaike’s Information Criterion (AIC), and Likelihood-Ratio Chi-square (LR χ^2^) statistics, degrees of freedom (df), and *p*-values were reported for main effects and interactions. Bonferroni-adjusted pairwise comparisons were conducted to evaluate differences among hosts and environmental conditions.

Flea survival data were analyzed separately using generalized linear models with a binomial distribution and logit link function. Blood meal host and environmental conditions were included as fixed effects. Model fit was evaluated using Likelihood-Ratio Chi-square (LR χ^2^) statistics, degrees of freedom (df), and *p*-values. Parameter estimates were interpreted relative to reference categories and reported as odds ratios (Exp (β)) with 95% confidence intervals where appropriate. Bonferroni-adjusted pairwise comparisons were conducted to evaluate differences among hosts and environmental conditions.

Crystal violet staining intensity data were quantified as absorbance values obtained from three independent biological replicates per environmental condition and incubation time point, with each biological replicate representing the mean of eight technical replicates from a single culture plate. Data were analyzed using GLMs with a Gamma distribution and log link function, including environmental condition and incubation time as fixed effects. Bonferroni-adjusted pairwise comparisons were used to evaluate differences among environmental conditions and incubation times. Significant effects were assessed using Likelihood-Ratio Chi-square (LR χ^2^) statistics, degrees of freedom (df), and *p*-values.

When applicable, data distributions were visualized using box plots or plots of biological replicate means with error bars representing ±1 standard deviation (SD). Outliers in box plots were defined as observations beyond 1.5× the interquartile range (IQR).

## 3. Results

### 3.1. Y. pestis Infection and Abiotic Factors Influence Blood Meal Digestion Rates of Rat Blood in X. cheopis

**Figure 1:**
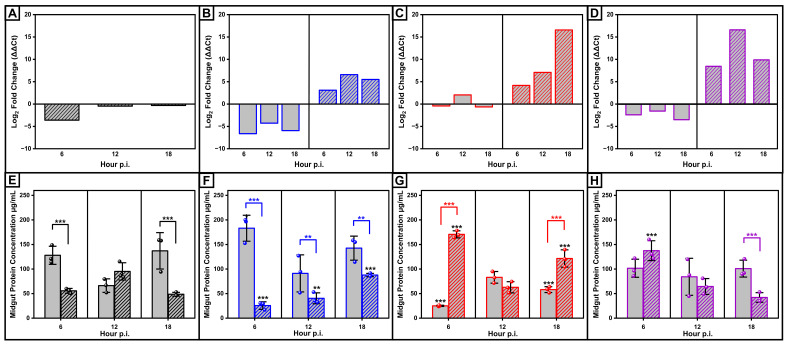
*Y. pestis* infection alters blood meal digestion and chymotrypsin expression in X. cheopis under varying environmental conditions. (**A**–**D**) Relative chymotrypsin expression normalized to *ef-1d* using the ΔΔCt method. Average Ct values from 3 independent biological replicates are shown as log_2_-transformed fold changes standardized to uninfected fleas under each condition. Each biological replicate consisted of mRNA prepared from dissected midguts pooled from 3 fleas and analyzed by qPCR in technical triplicate. (n = 9 fleas, 3 biological replicates per group). (**E**–**H**) Total protein concentrations in midgut lysates from uninfected (solid bars) and *Y. pestis-infected* (striped bars) fleas fed rat blood, measured at 6, 12, and 18 h post-feeding. Bars represent means of 3 independent biological replicates, error bars indicate ±1 SD, and individual points represent biological replicates. Statistical analysis: See the Materials and Methods Section for details. Colored brackets with asterisks indicate significant differences between uninfected and infected fleas within the same environmental condition and time point. Black asterisks above bars indicate significant differences relative to standard rearing conditions (70% RH, 25 °C) within the same infection status and time point. Only comparisons significantly different from standard rearing conditions are shown for clarity. Complete Bonferroni-adjusted pairwise comparisons are reported in Supplemental Table S1. Color code: 70% RH, 25 °C (black, **A**,**E**); 80% RH, 25 °C (blue, **B**,**F**); 70% RH, 30 °C (red, **C**,**G**); 80% RH, 30 °C (purple, **D**,**H**). ** *p* < 0.01, *** *p* < 0.001.

As a first approximation for understanding biotic and abiotic interactions with *Y. pestis* transmission by *X. cheopis*, we sought to characterize how rates of blood meal digestion responded to variation in species, temperature and relative humidity. Previously published work, however, typically involved qualitative assays such as midgut size and color to estimate digestion rates, which lack the sensitivity necessary for comparative statistics [58]. Nevertheless, these studies suggested that two peaks of activity over less than 24 h defined a typical digestion pattern for fleas. To assess this in *X. cheopis*, we carried out blood feeding with or without *Y. pestis* infection under standard rearing conditions, defined as 25 °C, 70% relative humidity and rat blood, and then quantified chymotrypsin-like gene expression using qPCR as a readout for active digestion over an 18 h time period. Under these conditions, uninfected fleas induced transcription of chymotrypsin at 6 h post-infection (HPI); however, lower expression was observed in *Y. pestis*-infected fleas (Figure 1A). This was surprising to us because previously published data, under slightly different environmental conditions of 21 °C/75% RH, showed an infection-related increase in chymotrypsin expression at 4 HPI [54]. To confirm this result with a different assay, we also measured total midgut protein levels over this time course. Similar to the chymotrypsin expression data, we found that total protein levels were lower in *Y. pestis*-infected midguts at 6 HPI (Figure 1E). When we shifted the environmental conditions, however, there was an infection-related increase in chymotrypsin expression (Figure 1B–D). In all cases, the levels of chymotrypsin expression in infected fleas remained consistent over the 18 h period. Total protein levels in the midgut typically exhibited a bimodal temporal response, with higher levels at 6 and 18 HPI compared to 12 HPI under most environmental conditions (Figure 1E–H).

We used GLM statistical analysis to assess the significance of the association at each time point. There was significance at 6 h (LR χ^2^ = 309.72, *p* < 0.001), 12 h (LR χ^2^ = 232.32, *p* < 0.001) and 18 h (LR χ^2^ = 283.42, *p* < 0.001), indicating that including environmental conditions, infection status, and their interaction significantly improved model fit relative to intercept-only models (Table 2). Species-specific models were also significant across these time points; however, the magnitude and direction of environmental and infection effects varied among hosts and over time. Significant pairwise comparisons highlighted host-specific differences in digestion rates among environmental conditions and demonstrated that infection variably altered these responses over time.

### 3.2. Abiotic Factors Influence the Dynamics of Y. pestis Infection in Rat-Fed X. cheopis

**Figure 2:**
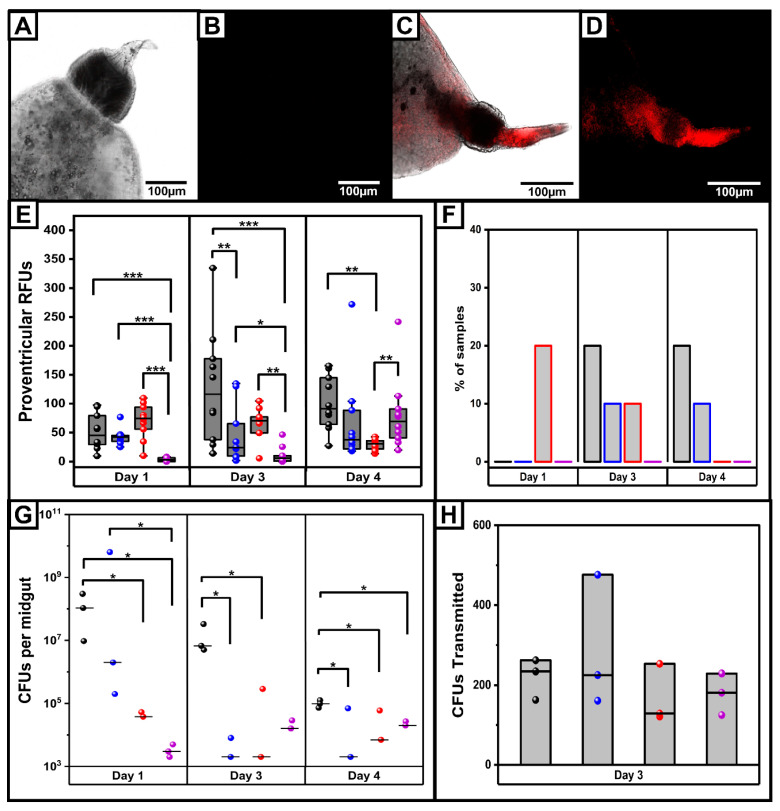
Environmental conditions influence *Y. pestis* aggregation in the esophagus and foregut of *X. cheopis.* (**A**–**D**) Representative confocal images: (**A**,**B**) uninfected flea; (**C**,**D**) *Y. pestis-*infected flea showing moderate-to-high fluorescence in the esophagus, classified as PIER. (**E**) Quantification of relative fluorescent units (RFUs) in the proventriculus on days 1, 3, and 4 post-infection; each data point represents 1 flea, with n = 10 per group and time point, collected in 3–5 independent trials. Box plots show 25–75% interquartile range (IQR) with whiskers for minimum and maximum values and a median line. (**F**) Percentage of fleas shown in E that exhibited PIER. (**G**) Colony-forming units (CFUs) recovered from infected fleas across days 1, 3, and 4 post-infection; each data point represents pooled midguts from 3 infected fleas, with 3 independent trials performed per condition (n = 9 fleas total per group). Horizontal lines indicate the median; samples with undetectable bacteria are not shown. (**H**) CFUs recovered following transmission assays on day 3 post-infection. Data shown were collected in 3 independent trials; each point represents one trial involving 1 group of 10 fleas. Bars indicate the maximum number of CFUs observed per condition; horizontal lines within bars represent the median. Statistical analysis: See the Materials and Methods Section for details. Asterisks indicate statistical significance compared to the groups indicated by each color; * *p* < 0.05, ** *p* < 0.01, *** *p* < 0.001. Color code: 70% RH, 25 °C (black); 80% RH, 25 °C (blue); 70% RH, 30 °C (red); 80% RH, 30 °C (purple).

During the first 7–14 days post-infection in fleas reared at 21 °C and 75% RH, *Y. pestis* has been reported to exist in bacterial aggregates in the midgut and foregut [59,60]. These masses appeared to change over time. For example, on days 1–3, *Y. pestis* was typically in multiple smaller aggregates, sometimes apparently free-floating in the midgut. Likewise, *Y. pestis* has also been reported in the esophagus [22]. Data suggest that these *Y. pestis* aggregates, especially those occurring in the esophagus prior to day 7 post-infection, could be indicative of transmission competency. We therefore employed a similar approach to assess the impact of temperature and humidity on EPT by quantifying *Y. pestis* aggregation in the midgut, foregut and esophagus during the first 7 days of infection (Supplemental Figure S1). Moderate-to-high proventricular fluorescence could be found in the midgut and proventriculus of infected fleas reared under standard rearing conditions (SRC) of 25 °C and 70% RH on days 1, 3, and 4 post-infection (Figure 2A–E). Up to 20% of fleas showed PIER on days 3 and 4 post-infection, whereas many harbored high proventricular but undetectable esophageal localization of *Y. pestis* (Figure 2E,F). We also quantified bacterial load in the midgut/foregut preparations by plating on YSA and found 10^6^–10^7^ CFU/midgut on days 1 and 3 post-infection, whereas on day 4, only 10^5^ CFU were recovered (Figure 2G). No detectable differences in transmission rates of fleas on day 3 post-infection were observed between any of the environmental conditions (Figure 2H). With GLM modeling of these data, we found that the environmental conditions significantly influenced proventricular RFU levels on days 1, 3 and 4 (positive results shown in Table 3 below). Therefore, aggregation of *Y. pestis* in the proventriculus may be sensitive to changes in the environment.

**Figure 3:**
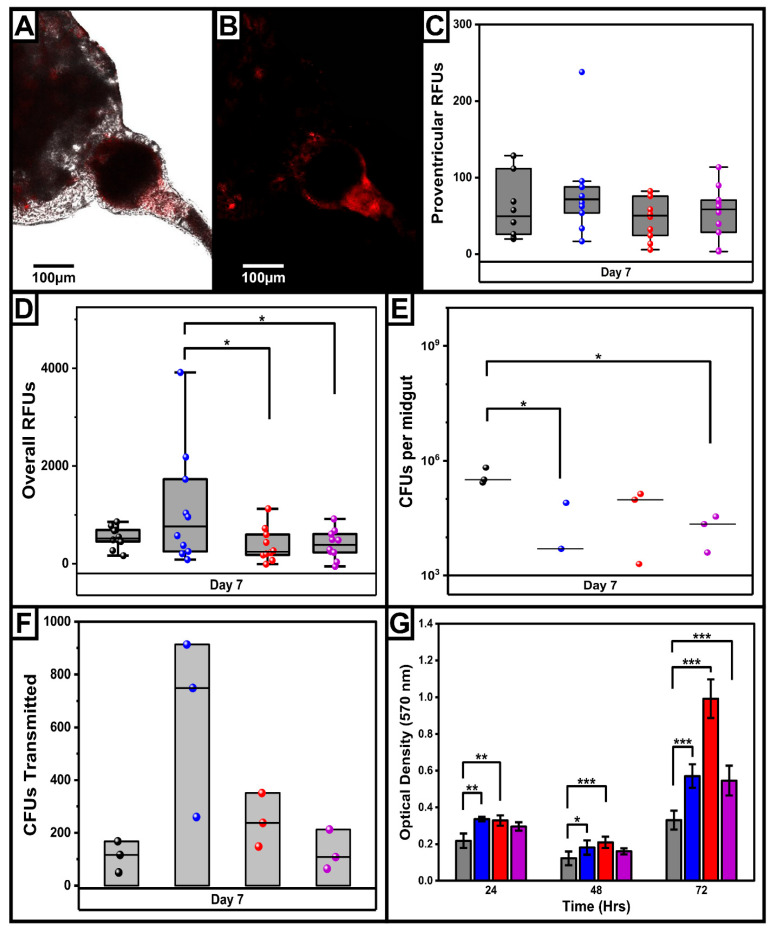
Increased humidity enhances biofilm-dependent transmission of *Y. pestis* by *X. cheopis*. (**A**,**B**) Representative confocal images of a flea midgut on day 7 post-infection showing increased dark masses associated with *Y. pestis*. (**C**) Proventricular RFU values in fleas infected with *Y. pestis* on day 7 post-infection; each data point represents 1 flea, with n = 10 per group and time point, collected in 3–5 independent trials. (**D**) Overall RFU values in the midguts shown in (**C**). (**C**,**D**) Box plots show 25–75% IQR with minimum and maximum values and a median line; outliers are shown. (**E**) CFUs in the midguts on day 7 post-infection; each data point represents pooled midguts from 3 infected fleas, with 3 independent trials performed per condition (n = 9 fleas total per group). Samples with no bacteria recovered are not shown; horizontal bars indicate the median number of CFUs. (**F**) CFUs recovered in transmission assays on day 7 post-infection. Data were collected in 3 independent trials; bars indicate the maximum number of CFUs observed; horizontal lines within bars represent the median. (**G**) Crystal violet staining of *Y. pestis* grown under the four environmental conditions. Bars represent means of 3 independent biological replicates (3 trials with 8 technical replicates each); error bars indicate ±1 SD. Only comparisons significantly different from standard rearing conditions are shown for clarity; complete Bonferroni-adjusted pairwise comparisons are reported in Supplemental Table S2. Statistical analysis: See the Materials and Methods Section for details. Color code: 70% RH, 25 °C (black); 80% RH, 25 °C (blue); 70% RH, 30 °C (red); 80% RH, 30 °C (purple). * *p* < 0.05, ** *p* < 0.01, *** *p* < 0.001.

This analysis was repeated, but the time point was changed to day 7 post-infection. Proventricular RFU levels were comparable across all environmental groups, with relatively low numbers of fleas with high RFU values or PIER, suggesting diffuse growth (Figure 3A–C). We also quantified overall midgut fluorescence to account for bacteria that may be encased in a midgut-localized mass. Using GLM analysis, we found that the environment had a significant impact on the overall RFU value but not on the proventricular RFU value (Figure 3D,E, summarized in Table 4 below). When assayed for transmission on day 7 post-infection, we observed no proventricular blockage post-feeding, suggesting low levels of biofilm. Nevertheless, bacteria were transmitted, and in fact, transmission appeared most efficient under the high humidity condition (Figure 3F). These day 7 results agree with day 3, suggesting a disconnect between *Y. pestis* aggregation in the proventriculus, esophagus or midgut and mass transmission rates.

There is some conflicting data on the thermal control over biofilm, with some reporting lower levels at higher temperature while others report higher levels at higher temperature. In order to gain a better understanding of how environmental factors influence extracellular polysaccharide/biofilm formation, we grew *Y. pestis* in culture under the defined conditions and used crystal violet staining of cultures to quantify biofilm. The highest level of crystal violet staining was observed at 72 h of incubation at high temperature (30 °C, 70% RH), whereas the lowest biofilm levels at 72 h were found under standard rearing conditions (25 °C, 70% RH) (Figure 3G). Under our experimental conditions, environment (LR χ^2^ = 56.85, *p* < 0.001), incubation time (LR χ^2^ = 103.90, *p* < 0.001), and their interaction (LR χ^2^ = 22.16, *p* = 0.001) significantly affected biofilm formation. Pairwise comparisons demonstrated increased biofilm formation at later time points, particularly under elevated temperature conditions. These data suggest that higher temperature may favor biofilm expression in culture.

### 3.3. Influence of Blood Host Species and Infection Status on Digestion Rates in X. cheopis

**Figure 4:**
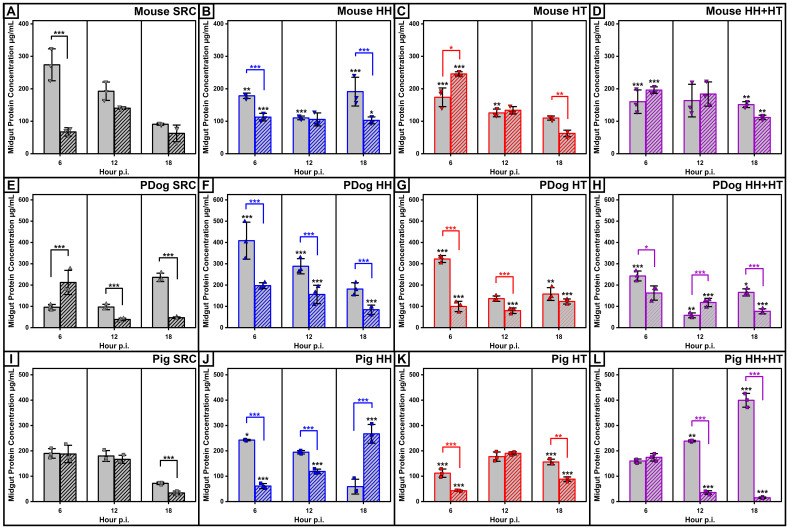
*Y. pestis* infection alters blood meal digestion in *X. cheopis* fleas in a host- and environment-dependent manner. (**A**–**D**) Total protein concentrations in midguts of fleas fed mouse blood, comparing uninfected (solid bars) and infected (striped bars) fleas at 6, 12, and 18 h post-feeding. (**E**–**H**) Same analysis for fleas fed prairie dog blood. (**I**–**L**) Same analysis for fleas fed pig blood. Bars represent means of 3 independent biological replicates, error bars indicate ±1 SD, and individual points represent biological replicates. Statistical analysis: See the Materials and Methods Section for details. Colored brackets with asterisks indicate significant differences between uninfected and infected fleas within the same environmental condition and time point. Black asterisks above bars indicate significant differences relative to standard rearing conditions (70% RH, 25 °C) within the same infection status and time point. Only comparisons significantly different from standard rearing conditions are shown for clarity; complete Bonferroni-adjusted pairwise comparisons are reported in Supplemental Table S1. Color code: 70% RH, 25 °C (black, **A**,**E**,**I**); 80% RH, 25 °C (blue, **B**,**F**,**J**); 70% RH, 30 °C (red, **C**,**G**,**K**); 80% RH, 30 °C (purple, **D**,**H**,**L**); * *p* < 0.05, ** *p* < 0.01, *** *p* < 0.001.

Since fleas typically feed on multiple animal hosts, we wondered if the impact of environmental factors on *Y. pestis* colonization of fleas was similar in its kinetics when fleas were fed from alternate hosts. We first assessed blood meal digestion under varying environmental conditions using different vertebrate host species’ blood, using the Bradford assay to measure total protein following midgut extraction. In fleas given sterile blood from mice, the patterns of digestion were similar to rats, with the infection apparently reducing digestion rates when fleas were housed at 25 °C but not at 30 °C, consistent with a thermally controlled host–pathogen interaction (Figure 4A–D). In contrast, however, this was not observed when the infection occurred in prairie dog or pig blood. With prairie dog blood, *Y. pestis* had an inhibitory effect on digestion rates compared to uninfected fleas across environmental conditions, except under SRC, where it appeared that infection actually increased the digestion rate (Figure 4E–H). In the case of pig blood, infection had no impact on digestion rates under SRC, but under high humidity or high temperature, it significantly reduced total protein at 6 HPI (Figure 4I–K). Under high humidity and high temperature, however, *Y. pestis* infection resulted in low midgut protein (Figure 4L). The GLM analysis demonstrated that environment, infection, and their interaction significantly influenced protein levels in the midgut for all three hosts, especially early in the digestion process (6 h post-feeding) (Table 2). These observations suggest that the environmental conditions influence gene expression in *Y. pestis* and in the flea, which may vary due to differences in blood chemistry between the vertebrate host species.

### 3.4. Influence of Blood Host Species on the Association of Abiotic Factors with Early-Phase Transmission of Y. pestis Infection

**Figure 5:**
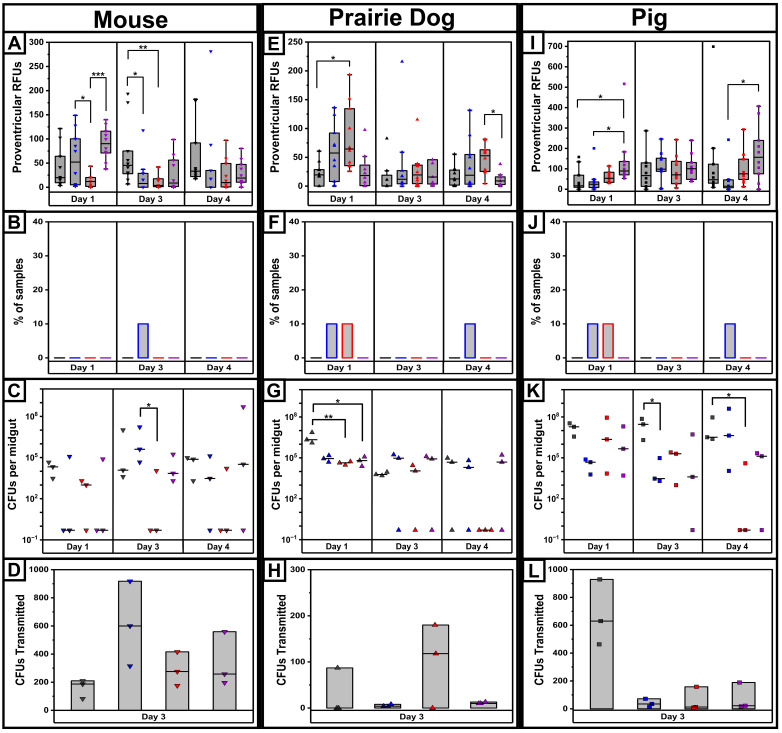
Host blood source influences esophageal aggregation and early-phase transmission in *X. cheopis* across environmental conditions. (**A**,**E**,**I**) Proventricular RFU values on days 1, 3, and 4 post-infection for fleas infected with mouse blood (**A**), prairie dog blood (**E**), and pig blood (**I**); each data point represents 1 flea, with n = 10 per group and time point, collected in 3–5 independent trials, except for prairie dog, under the following conditions: 70% RH, 25 °C (D3 n = 8, D4 n = 9) and 80% RH, 30 °C (D3 n = 7, D4 n = 9)). Box plots show 25–75% IQR with whiskers for minimum and maximum values and a median line; outliers are shown. (**B**,**F**,**J**) Percentage of fleas shown in (**A**,**E**,**I**) exhibiting PIER. (**C**,**G**,**K**) CFUs recovered from the midguts of infected fleas; each data point represents pooled midguts from 3 infected fleas, with 3 independent trials performed per condition (n = 9 fleas total per group). Horizontal bars indicate the median; samples with undetectable bacteria are shown below the detection threshold of 1 CFU. (**D**,**H**,**L**) CFUs recovered following transmission assays for fleas on day 3 post-infection. Each point represents one independent transmission assay (n = 3 independent assays per condition); bars indicate the maximum, and horizontal lines within each bar represent the median. Statistical analysis: See the Materials and Methods Section for details. Brackets with asterisks indicate significant differences between environmental conditions. Symbols: upside-down triangles: mouse, triangles: prairie dog, squares: pig. Color-code: 70% RH, 25 °C (black); 80% RH, 25 °C (blue); 70% RH, 30 °C (red); 80% RH, 30 °C (purple). * *p* < 0.05, ** *p* < 0.01, *** *p* < 0.001.

We also compared proventricular bacterial localization and PIER by microscopy to evaluate how host species’ blood source impacts the *Y. pestis* life cycle in the flea. Using mouse, prairie dog, or pig blood under the four environmental conditions, we quantified proventricular *Y. pestis* by microscopy on days 1, 3 and 4 post-infection. Infections using mouse blood showed environmental-condition-dependent differences, with the highest proventricular RFU levels on day 1 under conditions with higher heat and humidity (30 °C and 80% RH) (Figure 5A). However, PIER was not observed in this group and instead was most frequent under conditions of lower heat and higher humidity on day 3 post-infection (Figure 5B). There were apparent differences in bacterial growth for several conditions across time in the mouse-blood-infected fleas, indicating bacterial growth was also responsive to environmental conditions (Figure 5C). When assayed for transmission competency, the greatest median number of CFUs transmitted was in the group incubated at 25 °C and 80% RH (Figure 5D). Interestingly, the mouse-fed fleas housed at 30 °C, irrespective of RH, appeared to clear *Y. pestis* more efficiently, with more than 50% of fleas (combined from days 1, 3 and 4) harboring no bacteria. Overall, these results suggest that higher humidity may be more optimal for mouse-blood transmission.

While the mouse and rat blood gave similar results, prairie dog and pig blood were different. Colonization and PIER were low in fleas infected via prairie dog blood, suggesting diffuse bacterial growth (Figure 5E,F). Bacterial titers recovered from prairie-dog-fed fleas were not significantly impacted by the environmental conditions tested, except that a significantly higher titer of *Y. pestis* was recovered on day 1 post-infection under SRC compared to all other conditions and time points (Figure 5G). Given these data, perhaps it was to be expected that transmission on day 3 post-infection by prairie-dog-fed fleas appeared inefficient, with transmission detectable under high-temperature, low-humidity conditions, whereas no transmission was observed under the other conditions (Figure 5H). The combined data suggest that prairie dog blood poorly promotes EPT during *Y. pestis* infection of *X. cheopis*, but nonetheless, environmental factors still impacted bacterial growth in the flea.

In the pig model, the proventricular RFU values were relatively high at all time points and conditions (Figure 5I). Likewise, PIER was common, and the highest PIER frequency was observed in the higher heat and higher RH group on days 3 and 4 post-infection (Figure 5J). The bacterial loads for these times and conditions were detectable and were at the higher end but were lower than what was detected under SRC conditions, especially on day 4 (Figure 5K). Notably, on day 3 post-infection, SRC yielded the highest transmission efficiency in the artificial feeder assay, with significantly higher levels of bacteria transmitted compared to all other conditions (Figure 5L). Therefore, although PIER was most common under higher heat and RH conditions, this did not correlate with improved transmission in pig-blood-fed *X. cheopis*. GLM modeling of the different animal host species’ blood under the various environmental conditions indicated that environment influenced proventricular RFU values in a time- and species-dependent manner (Table 3). Combined with the transmission data, this suggests that proventricular localization of *Y. pestis*, in and of itself, is not an indicator that the flea is competent for transmission.

**Figure 6:**
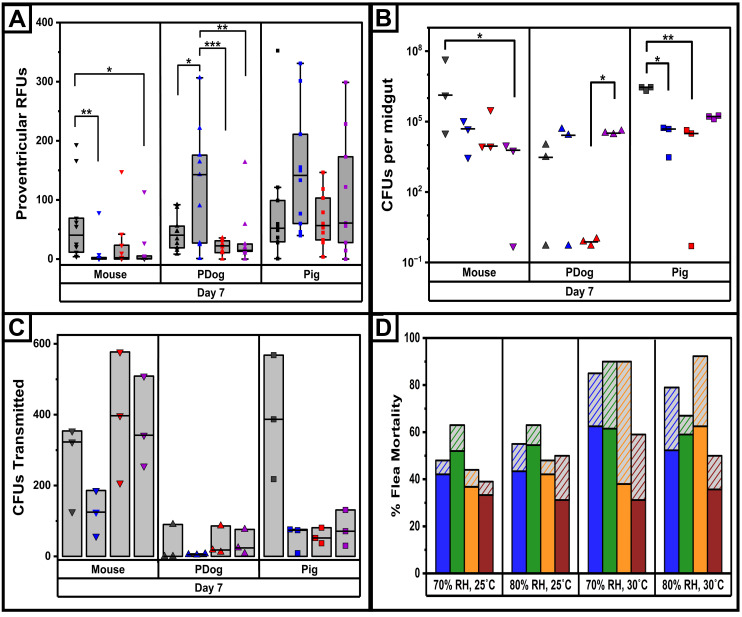
Environmental conditions and blood source shape *Y. pestis* infection and transmission in fleas on day 7 post-infection. (**A**) Proventricular RFU values on day 7 post-infection for *X. cheopis* infected with *Y. pestis* and fed different host blood sources. Each data point represents 1 flea (n = 10 per group, except for 80% RH, 30°C, which had n = 9 for all hosts); data shown were collected in 3–5 independent trials; box plots show 25–75% IQR with maximum and maximum values and a median line; outliers are shown. (**B**) CFUs from infected fleas on day 7 post-infection; each point represents pooled midguts from 3 infected fleas, with 3 independent trials performed per condition (n = 9 total fleas per group); horizontal bars indicate the median number of CFUs. (**C**) CFUs recovered following transmission on day 7 post-infection. Data were collected in 3 independent trials. Bars indicate the maximum number of CFUs, and horizontal lines within each bar represent the median. Color code: 70% RH, 25°C (black); 80% RH, 25°C (blue); 70% RH, 30°C (red); 80% RH, 30°C (purple). (**D**) Stacked columns showing flea mortality (%) on day 7 post-infection (solid portion) and day 14 post-infection (striped portion), stratified by host blood source (blue: rat; green: mouse; orange: prairie dog; maroon: pig); n = 33–52 per group. Statistical analysis: See the Materials and Methods Section for detailed description. Bonferroni-adjusted pairwise comparisons are shown in Supplemental Table S3. Brackets with asterisks indicate significant differences between environmental conditions; * *p* < 0.05, ** *p* < 0.01, *** *p* < 0.001.

This analysis was repeated, but the time point was changed to day 7 post-infection. *Y. pestis* transmission by mouse-fed fleas occurred under all environmental conditions on day 7 post-infection, though proventricular RFU values were low, suggesting no clear impact of the environment or proventricular colonization on transmission by mouse-fed fleas (Figure 6A,C). This differs from the transmission data collected on day 3 from mouse-fed fleas, and from the results in rat-fed fleas on day 7. Fleas infected with prairie dog blood had generally low proventricular fluorescence under SRC and other rearing conditions, although the RFU values were consistently highest when RH was high and lowest when the temperature was high on day 7 post-infection (Figure 6A). The low values in the latter group were mirrored in bacterial load, as we recovered no bacteria in the midguts of these fleas on day 7 (Figure 6B). However, these results were not mirrored in the RFU signals. Likewise, in spite of the seemingly robust growth of *Y. pestis* in prairie-dog-fed fleas on day 7 post-infection, the transmission assay again showed few to no bacteria were transmitted under any environmental condition. Therefore, there appeared to be no correlation between proventricular colonization, bacterial load, and transmission on day 7. The collective results suggest that in general, prairie dog blood poorly supports *Y. pestis* transmission by *X. cheopis*, at least in the early stage. Nevertheless, the GLM analysis reinforced the interactions between environment and proventricular RFU values in the prairie dog group on day 7 post-infection (Table 4).

The pig-fed fleas showed little response to temperature and humidity changes when proventricular RFU level or bacterial load was assessed, suggesting a high degree of aggregate-growth in the midgut on day 7 regardless of the temperature or humidity (Figure 6A,B). Environmental conditions for bacterial growth were optimal at SRC, with lower levels of *Y. pestis* recovered from the midgut of pig-fed fleas under all other conditions (Figure 6B). Likewise, bacterial transmission was significantly higher in pig-fed fleas housed under SRC than any other environmental condition (Figure 6C). These data suggest a correlation between bacterial load in the midgut and transmission efficiency on day 7 in pig-fed fleas. Moreover, this was the same result obtained from the day 3 transmission assays. Collectively, the data indicate significantly higher transmission levels in pig-fed fleas reared under SRC, with substantial loss of transmission under higher heat or humidity. As with the rat blood infections, we did not note the presence of proventricular blockage in any of the fleas used in the transmission assays, suggesting that D7 transmission is biofilm-independent (Figure 6C). Of note, flea mortality rates were relatively consistent across all blood meal host species on day 7 and day 14 post-infection, although pig-blood-fed fleas exhibited the lowest mortality under all conditions (Figure 6D). Higher temperature was associated with higher mortality on day 14 for the other animal blood sources. The GLM analysis indicated that pig blood increased survival probability at both time points relative to rat blood. In contrast, environmental effects emerged later in infection, significantly reducing survival by day 14 but not day 7, suggesting that environmental stressors exert cumulative impacts on flea survival over time. Due to the higher mortality rates under high temperature conditions, however, we were unable to evaluate D14 transmission or the potential impact of environmental conditions on biofilm formation. The combined data suggest that abiotic conditions may directly impact vector competence in a manner that is co-dependent on the animal source of the blood meal.

### 3.5. Associations Between Environment, Bacterial Aggregation and Growth Are Dependent on Host Species

**Figure 7:**
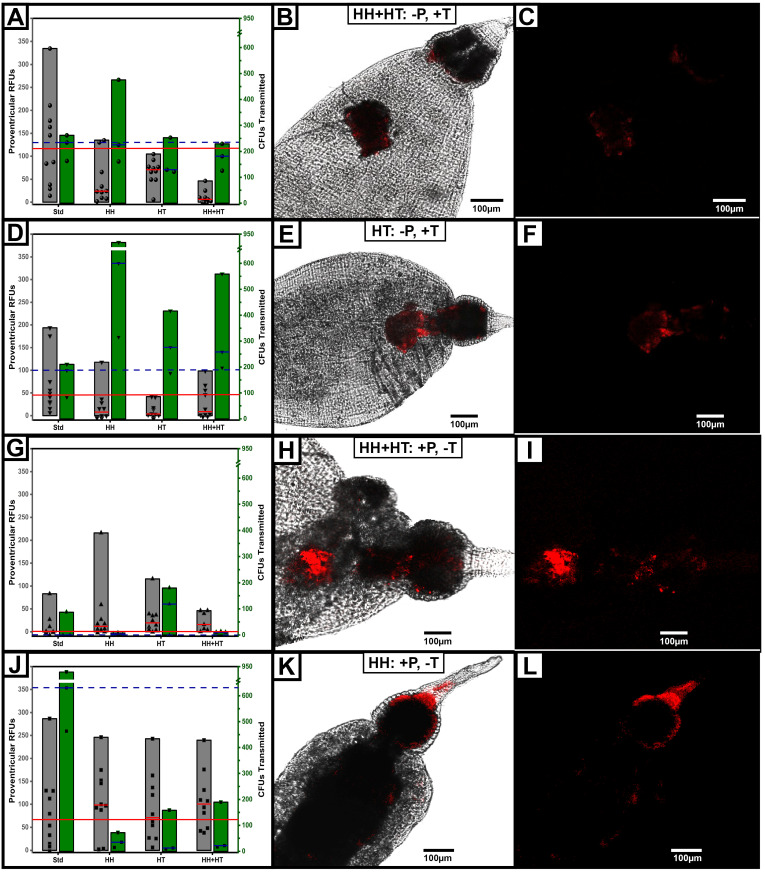
Environmental conditions and blood meal host modulate proventricular aggregation and transmission efficiency in *Xenopsylla cheopis*. Graphs represent a compilation of the data shown in Figure 2, Figure 3, Figure 5 and Figure 6 to assess correlation between mean proventricular fluorescence (gray bars) and bacteria (green bars) transmitted under each condition on day 3 post-infection. Rat (**A**–**C**), mouse (**D**–**F**), prairie dog (**G**–**I**), and pig (**J**–**L**) are shown. (**A**,**D**,**G**,**J**) Bars represent the maximum values, with individual data points shown to facilitate visualization of peak RFU values and transmission responses across environmental conditions. The red (RFU) or blue (CFU) dotted lines represent the median values observed under standard rearing conditions (SRC). (**B**,**C**) Representative confocal images of flea midguts on day 3 post-infection following feeding on rat blood under 80% RH and 30 °C (referred to as high humidity and high temperature (HH+HT)) show decreased proventricular RFU values compared to SRC (−P) but increased transmission compared to SRC (+T). Representative images are shown for fleas fed on mouse blood under 70%RH and 30 °C (HT) (**E**,**F**), prairie dog blood under HH+HT (**H**,**I**), and pig blood under 80% RH and 25 °C (HH). These examples illustrate the variable relationship between foregut colonization phenotypes and transmission efficiency across blood meal hosts and environmental conditions.

The predominant result from the data shown in Figure 2 and Figure 3 (rat-fed fleas) suggested a negative correlation between proventricular RFU intensity and transmission (Figure 7A). This is exemplified by the group incubated at 30 °C and 80% RH (high humidity (HH) + high temperature (HT)) on day 3 post-infection with low mean proventricular fluorescence (−P) yet higher levels of bacteria transmitted (+T) (Figure 7B,C). In fleas infected with mouse blood, a lower mean proventricular RFU level was also associated with higher transmission of *Y. pestis* on day 3, exemplified best by the high-temperature (HT), low-RH group (Figure 7D–F). These data may suggest that reduced aggregation on the proventriculus is more conducive to EPT. Consistent with this interpretation, in prairie-dog- or pig-fed fleas, an increase in proventricular RFU levels actually correlated with lower transmission levels, and this was exemplified in two groups: 25 °C, 80% RH (high humidity (HH)) and HH+HT groups (Figure 7G–L). Overall, these data suggest that the formation of aggregates in the flea midgut during early infection is influenced by environmental factors and the host species. Moreover, the collective observations suggest that diffusely localized *Y. pestis* is competent for EPT in *X. cheopis*.

## 4. Discussion

*Xenopsylla cheopis* was identified as a highly efficient plague vector due to its abundance during the pandemics and its tendency to develop proventricular blockage during *Y. pestis* infection [61]. However, many other flea species have been found to be highly efficient plague vectors with varying levels of proventricular blockage during *Y. pestis* infection [8,62]. In contrast, most, if not all, flea species are competent for early-phase/mass transmission of *Y. pestis*, a mechanism that efficiently operates during the first 1–7 days following infection. As these rapid kinetics are more in line with the rates of epidemic spread of plague, the early-phase mechanism is thought to make critical contributions to the sylvatic plague cycle [13].

In this work, we investigated whether environmental factors (temperature and humidity) and host blood source directly influence *Y. pestis* infection dynamics and early-phase transmission competence of *X. cheopis*. In agreement with published data, we found that environmental effects on digestion and *Y. pestis* infection dynamics in fleas are strongly dependent on host blood source. Digestion assays showed large interaction effects between environment and infection status, with a possible temperature-dependent impact. Host-specific analyses revealed significant environmental impacts on bacterial load and EPT efficiency, suggesting that physiological or biochemical factors modulate how the environment shapes pathogen persistence and growth in fleas. The results indicate that the vector–pathogen–environment relationships cannot be generalized across hosts, and that transmission potential may be especially sensitive to environmental variation in certain hosts.

Significant interaction effects between environment, infection and midgut protein levels indicate that host–pathogen interactions in the midgut alter normal physiological responses to feeding. The most dominant influence was temperature, with the GLM model suggesting both fleas and bacteria have differential responses dependent on the environmental temperature. Furthermore, we demonstrated that the vertebrate host species’ blood influences these interactions, which may yield important differences in bacterial clearance or growth. For example, prairie-dog-fed fleas were significantly more effective at clearing the infection under HH conditions compared to the other conditions, with lower transmission rates. Although the interpretation of this result could be confounded by the fact that the prairie dog is not a native host of *X. cheopis*, it may also indicate that the abundance of vertebrate hosts that may stimulate improved immune responses could potentially slow the spread of disease regardless of environmental changes.

Although our data quantifying *Y. pestis* aggregation in the midgut and proventriculus using fluorescence microscopy were largely in agreement with published data collected with GFP-expressing *Y. pestis*, there were dependencies on the environment that may influence data interpretation. All of our assays occurred in the first 7 days of infection, during which time there is little evidence suggesting the fleas have developed competency for biofilm-dependent transmission. Nevertheless, we found variable correlations between *Y. pestis* aggregation in the midgut, proventriculus, or esophagus and transmission. Although bacterial load in the midgut appeared to consistently correlate with transmission, notable negative correlations with increased humidity were observed at different time points and across experimental conditions. The collective results suggest that *Y. pestis* aggregation is not a prerequisite for EPT.

Our findings indicate a dynamic system in which temperature, humidity, and host blood interact to shape *Y. pestis* infection and transmission in *X. cheopis*. Considering the highly variable and species-specific responses, predictive mathematical models of the zoonotic plague cycle could be modified to account for these interactions [13,32,63]. This is particularly important in the face of changes that impact climate and land usage, which may alter burrow microenvironments and/or shift food source availability and animal populations in ways that might change the transmission dynamics. While this study focused on a single flea species under controlled feeding conditions, over 200 rodent species are hosts for *Y. pestis*, with 30 known flea vectors living in varying habitats throughout the world [64]. It may therefore be some time before we have enough data to define unifying trends of *Y. pestis* transmission in natural systems.

## Figures and Tables

**Table 1:** Primers for elongation factor 1D, used as the housekeeping gene in qPCR analysis and primers designed for chymotrypsin-like serine protease in *Ctenocephalides felis*, GenBank accession AF053912.1.

Gene	Forward	Reverse
*ef-1d*	5′-ACTCTTGTTGAACCAGCCCG-3′	5′-GGTTACGGTTACAGCATCAGTG-3′
*CfSP*	5′-ATTCGTTGAGCCAACTTCGC-3′	5′-AACGAAGAAACGCCAACCAG-3′

**Table 2:** Digestion dynamics were significantly influenced by host blood, environmental conditions, and infection status, both independently and through their interactions.

Six Hours of Digestion				
	Effect	LR χ^2^	df	*p*-Value
All Species	Host	194.4	3	<0.001
	Enviro	68.7	3	<0.001
	Infection	106.9	1	<0.001
	Host * Enviro	151.9	9	<0.001
	Host * Infection	29.9	3	<0.001
	Enviro * Infection	148.3	3	<0.001
	Host * Enviro * Infection	230.2	9	<0.001
	Model Fit: LR χ^2^ = 309.72, df = 31, *p* < 0.001, AIC = 878.34.			
Rat	Effect	LR χ^2^	df	*p*-Value
	Enviro	34.8	3	<0.001
	Infection	6.3	1	0.012
	Enviro * Infection	80.6	3	<0.001
	Model Fit: LR χ^2^ = 81.48, df = 7, *p* < 0.001, AIC = 200.19.			
Mouse	Enviro	27.3	3	<0.001
	Infection	25.9	1	<0.001
	Enviro * Infection	53.9	3	<0.001
	Model Fit: LR χ^2^ = 60.34, df = 7, *p* < 0.001, AIC = 227.84.			
Prairie Dog	Enviro	33.1	3	<0.001
	Infection	23.4	1	<0.001
	Enviro * Infection	46.9	3	<0.001
	Model Fit: LR χ^2^ = 58.99, df = 7, *p* < 0.001, AIC = 246.65.			
Pig	Enviro	68.7	3	<0.001
	Infection	56.3	1	<0.001
	Enviro * Infection	60.4	3	<0.001
	Model Fit: LR χ^2^ = 85.02, df = 7, *p* < 0.001, AIC = 204.28.			
Twelve Hours of Digestion				
	Effect	LR χ^2^	df	*p*-Value
All Species	Host	136	3	<0.001
	Enviro	18.1	3	<0.001
	Infection	63.3	1	<0.001
	Host * Enviro	134.9	9	<0.001
	Host * Infection	31.5	3	<0.001
	Enviro * Infection	12.7	3	0.005
	Host * Enviro * Infection	126.1	9	<0.001
	Model Fit: LR χ^2^ = 232.32, df = 31, *p* < 0.001, AIC = 888.33.			
Rat	Effect	LR χ^2^	df	*p*-Value
	Enviro	3.7	3	0.298
	Infection	5.8	1	0.016
	Enviro * Infection	13.5	3	0.004
	Model Fit: LR χ^2^ = 17.94, df = 7, *p* = 0.012, AIC = 221.25.			
Mouse	Enviro	27.2	3	<0.001
	Infection	0.7	1	0.402
	Enviro * Infection	8.2	3	0.041
	Model Fit: LR χ^2^ = 30.79, df = 7, *p* < 0.001, AIC = 225.6.			
Prairie Dog	Enviro	60.2	3	<0.001
	Infection	21.1	1	<0.001
	Enviro * Infection	41.6	3	<0.001
	Model Fit: LR χ^2^ = 69.5, df = 7, *p* < 0.001, AIC = 214.96.			
Pig	Enviro	56.7	3	<0.001
	Infection	62.9	1	<0.001
	Enviro * Infection	74.2	3	<0.001
	Model Fit: LR χ^2^ = 84.89, df = 7, *p* < 0.001, AIC = 205.97.			
Eighteen Hours of Digestion				
	Effect	LR χ^2^	df	*p*-Value
All Species	Host	61.5	3	<0.001
	Enviro	86.9	3	<0.001
	Infection	160	1	<0.001
	Host * Enviro	82.9	9	<0.001
	Host * Infection	31.7	3	<0.001
	Enviro * Infection	131.3	3	<0.001
	Host * Enviro * Infection	194.9	9	<0.001
	Model Fit: LR χ^2^ = 283.42, df = 31, *p* < 0.001, AIC = 852.83.			
Rat	Effect	LR χ^2^	df	*p*-Value
	Enviro	25.7	3	<0.001
	Infection	27.9	1	<0.001
	Enviro * Infection	46.9	3	<0.001
	Model Fit: LR χ^2^ = 57.31, df = 7, *p* < 0.001, AIC = 203.97.			
Mouse	Enviro	32	3	<0.001
	Infection	26.9	1	<0.001
	Enviro * Infection	3.7	3	0.298
	Model Fit: LR χ^2^ = 43.58, df = 7, *p* < 0.001, AIC = 220.9.			
Prairie Dog	Enviro	12.9	3	0.005
	Infection	60.4	1	<0.001
	Enviro * Infection	37.5	3	<0.001
	Model Fit: LR χ^2^ = 66.44, df = 7, *p* <0.001, AIC = 216.01.			
Pig	Enviro	40.8	3	<0.001
	Infection	43.2	1	<0.001
	Enviro * Infection	75.4	3	<0.001
	Model Fit: LR χ^2^ = 86.23, df = 7, *p* < 0.001, AIC = 215.19.			

Note: * represents the interaction between the main factors.

**Table 3:** Blood meal host species and blood meal host and environmental interactions were significant factors in proventricular colonization, while environmental conditions alone did not have a consistently significant effect.

Day 1 Post-Infection							
	Effect	LR χ^2^	*p*-Value		Effect	LR χ^2^	*p*-Value
All Species	Host	18.6	<0.001	Rat	Enviro	44.5	<0.001
	Enviro	5.7	0.125	Model Fit: df = 3, AIC = 335.46			
	Host * Enviro	79.5	<0.001	Mouse	Enviro	21.7	<0.001
Model Fit: LR χ^2^ = 92.54, df = 15, *p* < 0.001, AIC = 1531.43				Model Fit: df = 3, AIC = 389.38			
				Prairie Dog	Enviro	13.4	0.004
				Model Fit: df = 3, AIC = 375.9			
				Pig	Enviro	15.4	0.002
				Model Fit: df = 3, AIC = 424.53			
Day 3 Post-Infection							
	Effect	LR χ^2^	*p*-Value		Effect	LR χ^2^	*p*-Value
All Species	Host	67.7	<0.001	Rat	Enviro	35.1	<0.001
	Enviro	10.9	0.012	Model Fit: df = 3, AIC = 400.09			
	Host * Enviro	43.9	<0.001	Mouse	Enviro	17.3	<0.001
Model Fit: LR χ^2^ = 107.19, df = 15, *p* < 0.001, AIC = 1551.45				Model Fit: df = 3, AIC = 373.6			
				Prairie Dog	Enviro	3.2	0.360
				Model Fit: df = 3, AIC = 326.38			
				Pig	Enviro	0.86	0.836
				Model Fit: df = 3, AIC = 455.67			
Day 4 Post-Infection							
	Effect	LR χ^2^	*p*-Value		Effect	LR χ^2^	*p*-Value
All Species	Host	53.2	<0.001	Rat	Enviro	16.3	0.001
	Enviro	4.3	0.232	Model Fit: df = 3, AIC = 412.49			
	Host * Enviro	38.1	<0.001	Mouse	Enviro	7.5	0.57
Model Fit: LR χ^2^ = 94.99, df = 15, *p* < 0.001, AIC = 1638.67				Model Fit: df = 3, AIC = 481.99			
				Prairie Dog	Enviro	14.1	0.003
				Model Fit: df = 3, AIC = 341.76			
				Pig	Enviro	11.5	0.009
				Model Fit: df = 3, AIC = 472.31			

Note: * represents the interaction between the main factors. For All Species: Host and Enviro df = 3; Host * Enviro df = 9. Species-specific: Enviro df = 3.

**Table 4:** Blood meal host species had significant effects on proventricular and overall RFU values on day 7 post-infection, whereas environmental effects were limited but showed significant host interactions.

Day 7 Proventricular RFUs							
	Effect	LR χ^2^	*p*-Value		Effect	LR χ^2^	*p*-Value
All Species	Host	57.8	<0.001	Rat	Enviro	4.2	0.242
	Enviro	12.9	0.005	Model Fit: df = 3, AIC = 408.27			
	Host * Enviro	37.1	<0.001	Mouse	Enviro	18.3	<0.001
Model Fit: LR χ^2^ = 96.65, df = 15, *p* < 0.001, AIC = 1595.57				Model Fit: df = 3, AIC = 359.07			
				Prairie Dog	Enviro	26.8	<0.001
				Model Fit: df = 3, AIC = 380.22			
				Pig	Enviro	6.2	0.101
				Model Fit: df = 3, AIC = 448.29			
Day 7 Overall RFUs							
	Effect	LR χ^2^	*p*-Value		Effect	LR χ^2^	*p*-Value
All Species	Host	106.3	<0.001	Rat	Enviro	14.2	0.003
	Enviro	12.8	0.005	Model Fit: df = 3, AIC = 598.4			
	Host * Enviro	47.2	<0.001	Mouse	Enviro	28.9	<0.001
Model Fit: LR χ^2^ = 132.89, df = 15, *p* < 0.001, AIC = 2307.6				Model Fit: df = 3, AIC = 515.16			
				Prairie Dog	Enviro	15.2	0.002
				Model Fit: df = 3, AIC = 561.15			
				Pig	Enviro	1.8	0.621
				Model Fit: df = 3, AIC = 638.48			

Note: * represents the interaction between the main factors. For All Species: Host and Enviro df = 3; Host * Enviro df = 9. Species-specific: Enviro df = 3.

## Data Availability

The raw data supporting the conclusions of this article are available in the Supplementary Information folder.

## Supplementary Materials

The following supporting information can be downloaded at: https://www.mdpi.com/article/10.3390/pathogens15060639/s1, Figure S1: Visualization of proventricular colonization and biofilm formation using confocal microscopy to detect bacteria carrying tdTomato. Table S1: Statistical analysis of data quantifying total midgut protein levels showing significant Bonferroni-adjusted pairwise comparisons across environmental conditions, host species, and time for fleas fed on *Y. pestis*-infected or sterile/uninfected blood. Table S2: Statistical analysis of data quantifying crystal violet staining showing significant Bonferroni-adjusted pairwise comparisons across environmental conditions and time. Table S3: Statistical analysis of survival data of *Y. pestis*-infected fleas showing significant Bonferroni-adjusted pairwise comparisons across host species, environmental conditions and time.

## Abbreviations

The following abbreviations are used in this manuscript:

EPT	Early-phase transmission
PIER	Post-infectious esophageal reflux
HIA	Heart infusion agar
YSA	Yersinia selective agar
RH	Relative humidity
MIN	Minutes
CFUs	Colony-forming units
RFUs	Relative fluorescent units
GLM	Generalized linear model
HPI	Directory of open access journals
SRC	Standard rearing conditions
HH	High humidity
HT	High temperature
