# Supplementary material for: Environmental and Host Blood Interactions Shape Yersinia pestis Dynamics in the Rat Flea, Xenopsylla cheopis
Source: Pathogens. 2026 Jun 16;15(6):639. doi: 10.3390/pathogens15060639 (PMC13305545; doi:10.3390/pathogens15060639)
Supplement: Supplementary file 1 [file pathogens-15-00639-s001.zip › pathogens-4269821-supplementary.pdf]

Supplemental Information.

Environmental and host blood interactions shape Yersinia pestis dynamics in the rat flea, Xenopsylla cheopis

Pauling, CD and Anderson, DM

File contains 3 tables and 1 figure.

Supplemental Table S1. Statistical analysis of data quantifying total midgut protein levels showing significant Bonferroni-adjusted pairwise comparisons across environmental conditions, host species, and time for fleas fed on *Y. pestis*-infected or sterile/uninfected blood.

		**Rat**			
**6 Hours Uninfected**	**Comparison**	**Mean Difference**	**Std. Error**	**p-value**	**Interpretation[A]**
	SRC vs HH	-55.18	17.10	0.035	SRC < HH
	SRC vs HT	102.93	9.98	<0.001	SRC > HT
	HH vs HT	158.12	14.15	<0.001	HH > HT
	HH vs HH+HT	81.58	16.03	<0.001	HH > HH+HT
	HT vs HH+HT	-76.53	8.01	<0.001	HT < HH+HT
**6 Hours Infected**	**Comparison**	**Mean Difference**	**Std. Error**	**p-value**	**Interpretation[A]**
	SRC vs HH	30.0	4.69	<0.001	SRC > HH
	HH vs HT	-144.99	13.20	<0.001	HH < HT
	HH vs HH+HT	-111.77	10.69	<0.001	HH < HH+HT
**12 Hours Uninfected**	**Comparison**	**Mean Difference**	**Std. Error**	**p-value**	**Interpretation[A]**
		All pairwise comparisons were non-significant			
**12 Hours Infected**	**Comparison**	**Mean Difference**	**Std. Error**	**p-value**	**Interpretation[A]**
	SRC vs HH	54.78	14.28	0.004	SRC > HH
**18 Hours Uninfected**	**Comparison**	**Mean Difference**	**Std. Error**	**p-value**	**Interpretation[A]**
	SRC vs HT	78.95	11.99	<0.001	SRC > HT
	HH vs HT	84.42	12.39	<0.001	HH > HT
	HT vs HH+HT	-42.73	9.37	<0.001	HT < HH+HT
**18 Hours Infected**	**Comparison**	**Mean Difference**	**Std. Error**	**p-value**	**Interpretation[A]**
	SRC vs HH	-39.06	8.08	<0.001	SRC < HH
	SRC vs HT	-72.84	10.54	<0.001	SRC < HT
	HH vs HH+HT	45.61	7.84	<0.001	HH > HH+HT
	HT vs HH+HT	79.39	10.36	<0.001	HT > HH+HT
		**Mouse**			
**6 Hours Uninfected**	**Comparison**	**Mean Difference**	**Std. Error**	**p-value**	**Interpretation[A]**
	SRC vs HH	95.53	22.29	0.001	SRC > HH
	SRC vs HT	100.07	22.13	<0.001	SRC > HT
	SRC vs HH+HT	113.60	21.64	<0.001	SRC > HH+HT
**6 Hours Infected**	**Comparison**	**Mean Difference**	**Std. Error**	**p-value**	**Interpretation[A]**
	SRC vs HH	-45.53	8.95	<0.001	SRC < HH
	SRC vs HT	-178.67	17.38	<0.001	SRC < HT
	SRC vs HH+HT	-128.80	14.13	<0.001	SRC < HH+HT
	HH vs HT	-133.13	18.44	<0.001	HH < HT
	HH vs HH+HT	-83.27	15.42	<0.001	HH < HH+HT
**12 Hours Uninfected**	**Comparison**	**Mean Difference**	**Std. Error**	**p-value**	**Interpretation[A]**
	SRC vs HH	82.13	16.66	<0.001	SRC > HH
	SRC vs HT	66.80	17.26	0.003	SRC > HT
	HH vs HH+HT	-53.33	14.83	0.009	HH < HH+HT
**12 Hours Infected**	**Comparison**	**Mean Difference**	**Std. Error**	**p-value**	**Interpretation[A]**
	HH vs HH+HT	-78.27	15.93	<0.001	HH < HH+HT
**18 Hours Uninfected**	**Comparison**	**Mean Difference**	**Std. Error**	**p-value**	**Interpretation[A]**
	SRC vs HH	-100.40	19.77	<0.001	SRC < HH
	SRC vs HH+HT	-60.40	16.48	0.007	SRC < HH+HT
	HH vs HT	81.40	20.59	0.002	HH > HT
**18 Hours Infected**	**Comparison**	**Mean Difference**	**Std. Error**	**p-value**	**Interpretation[A]**
	SRC vs HH	-40.07	11.24	0.010	SRC < HH
	SRC vs HH+HT	-49.07	11.97	0.001	SRC < HH+HT
	HH vs HT	40.27	11.23	0.009	HH > HT
	HT vs HH+HT	-49.27	11.96	0.001	HT < HH+HT
		**Prairie Dog**			
**6 Hours Uninfected**	**Comparison**	**Mean Difference**	**Std. Error**	**p-value**	**Interpretation[A]**
	SRC vs HH	-312.58	35.51	<0.001	SRC < HH
	SRC vs HT	-226.52	28.46	<0.001	SRC < HT
	SRC vs HH+HT	-146.80	22.08	<0.001	SRC < HH+HT
	HH vs HH+HT	165.78	40.21	0.001	HH > HH+HT
**6 Hours Infected**	**Comparison**	**Mean Difference**	**Std. Error**	**p-value**	**Interpretation[A]**

	SRC vs HT	112.80	19.84	<0.001	SRC > HT
	HH vs HT	97.78	18.69	<0.001	HH > HT
	HT vs HH+HT	-63.13	16.13	0.003	HT < HH+HT
**12 Hours Uninfected**	**Comparison**	**Mean Difference**	**Std. Error**	**p-value**	**Interpretation[A]**
	SRC vs HH	-191.28	25.21	<0.001	SRC < HH
	SRC vs HH+HT	39.14	9.36	0.001	SRC > HH+HT
	HH vs HT	151.93	26.43	<0.001	HH > HT
	HH vs HH+HT	230.42	24.37	<0.001	HH > HH+HT
	HT vs HH+HT	78.49	12.27	<0.001	HT > HH+HT
**12 Hours Infected**	**Comparison**	**Mean Difference**	**Std. Error**	**p-value**	**Interpretation[A]**
	SRC vs HH	-117.34	13.30	<0.001	SRC < HH
	SRC vs HT	-41.14	7.33	<0.001	SRC < HT
	SRC vs HH+HT	-79.57	10.29	<0.001	SRC < HH+HT
	HH vs HT	76.20	14.50	<0.001	HH > HT
	HT vs HH+HT	-38.42	11.80	0.032	HT < HH+HT
**18 Hours Uninfected**	**Comparison**	**Mean Difference**	**Std. Error**	**p-value**	**Interpretation[A]**
	SRC vs HT	78.41	20.66	0.004	SRC > HT
	SRC vs HH+HT	70.57	20.98	0.022	SRC > HH+HT
**18 Hours Infected**	**Comparison**	**Mean Difference**	**Std. Error**	**p-value**	**Interpretation[A]**
	SRC vs HH	-37.61	7.01	<0.001	SRC < HH
	SRC vs HT	-76.20	9.57	<0.001	SRC < HT
	SRC vs HH+HT	-29.94	6.53	<0.001	SRC < HH+HT
	HH vs HT	-38.59	10.84	0.010	HH < HT
	HT vs HH+HT	46.26	10.54	<0.001	HT > HH+HT
		**Pig**			
**6 Hours Uninfected**	**Comparison**	**Mean Difference**	**Std. Error**	**p-value**	**Interpretation[A]**
	SRC vs HH	-52.58	16.38	0.037	SRC < HH
	SRC vs HT	77.92	11.72	<0.001	SRC > HT
	HH vs HT	130.50	14.20	<0.001	HH > HT
	HH vs HH+HT	82.50	15.45	<0.001	HH > HH+HT
	HT vs HH+HT	-48.00	10.39	<0.001	HT < HH+HT
**6 Hours Infected**	**Comparison**	**Mean Difference**	**Std. Error**	**p-value**	**Interpretation[A]**
	SRC vs HH	126.43	10.49	<0.001	SRC > HH
	SRC vs HT	144.90	10.23	<0.001	SRC > HT
	HH vs HT	18.47	3.97	<0.001	HH > HT
	HH vs HH+HT	-113.27	9.83	<0.001	HH < HH+HT
	HT vs HH+HT	-131.73	9.55	<0.001	HT < HH+HT
**12 Hours Uninfected**	**Comparison**	**Mean Difference**	**Std. Error**	**p-value**	**Interpretation[A]**
	SRC vs HH+HT	-58.75	14.45	0.001	SRC < HH+HT
	HT vs HH+HT	-61.33	14.38	0.001	HT < HH+HT
**12 Hours Infected**	**Comparison**	**Mean Difference**	**Std. Error**	**p-value**	**Interpretation[A]**
	SRC vs HH	47.73	9.89	<0.001	SRC > HH
	SRC vs HH+HT	130.88	8.24	<0.001	SRC > HH+HT
	HH vs HT	-71.35	10.85	<0.001	HH < HT
	HH vs HH+HT	83.15	6.00	<0.001	HH > HH+HT
	HT vs HH+HT	154.50	9.36	<0.001	HT > HH+HT
**18 Hours Uninfected**	**Comparison**	**Mean Difference**	**Std. Error**	**p-value**	**Interpretation[A]**
	SRC vs HT	-84.25	17.01	<0.001	SRC < HT
	SRC vs HH+HT	-327.58	40.17	<0.001	SRC < HH+HT
	HH vs HT	-97.25	16.51	<0.001	HH < HT
	HH vs HH+HT	-340.58	39.96	<0.001	HH < HH+HT
	HT vs HH+HT	-243.33	42.45	<0.001	HT < HH+HT
**18 Hours Infected**	**Comparison**	**Mean Difference**	**Std. Error**	**p-value**	**Interpretation[A]**
	SRC vs HH	-233.12	26.65	<0.001	SRC < HH
	SRC vs HT	-54.45	9.38	<0.001	SRC < HT
	SRC vs HH+HT	18.90	3.68	<0.001	SRC > HH+HT
	HH vs HT	178.68	27.85	<0.001	HH > HT
	HH vs HH+HT	252.03	26.48	<0.001	HH > HH+HT
	HT vs HH+HT	73.35	8.88	<0.001	HT > HH+HT

A: environmental conditions and abbreviations: SRC (25°C, 70% relative humidity (RH)); HH (25°C, 80% RH); HT (30°C, 70% RH); HH+HT (30°C, 80% RH).

Supplemental Table S2. Statistical analysis of data quantifying crystal violet staining showing significant Bonferroni-adjusted pairwise comparisons across environmental conditions and time.

Biofilm				
**All Timepoints**	**Effect**	**LR χ^2**	**df**	**p-value**
	Enviro	58.85	3	<0.001
	Hour	103.90	2	<0.001
	Hour*Enviro	22.16	6	0.001
* Model Fit: LR χ^2 = 115.89, df = 11, p <0.001, AIC = -109.18				
**Pairwise Comparison**				
**24 Hours**	**Comparison**	**Mean Difference**	**Std. Error**	**p-value**
	SRC vs HH	0.12	0.03	0.003
	SRC vs HT	-0.11	0.28	0.006
**48 Hours**	**Comparison**	**Mean Difference**	**Std. Error**	**p-value**
	SRC vs HH	0.06	0.02	0.011
	SRC vs HT	-0.09	0.02	<0.001
**48 Hours**	**Comparison**	**Mean Difference**	**Std. Error**	**p-value**
	SRC vs HH	0.24	0.05	<0.001
	SRC vs HT	-0.66	0.07	<0001
	SRC vs HH+HT	-0.22	0.05	<0.001
	HH vs HT	-0.42	0.08	<0.001
	HT vs HH+HT	0.45	0.08	<0.001

Supplemental Table S3. Statistical analysis of survival data of *Y. pestis*-infected fleas showing significant Bonferroni-adjusted pairwise comparisons across host species, environmental conditions and time.

**Day 7 Flea Mortality**						
**Effect**	**LR χ^2**	**p-value**		**Host**	**Exp (β)[A]**	**p-value**
Host	9.47	0.024	**Bloodmeal**	Mouse	0.792	0.426
Enviro	3.42	0.332		Prairie Dog	1.268	0.435
* Model Fit: LR χ^2 = 12.80, df = 6, p=0.046, AIC = 73.11				Pig	2.153	0.026
				**Condition**	**Exp (β)[A]**	**p-value**
			**Enviro**	HH	0.926	0.812
				HT	0.689	0.231
				HH+HT	0.602	0.116
**Day 14 Flea Mortality**						
**Effect**	**LR χ^2**	**p-value**		**Host**	**Exp (β)[A]**	**p-value**
Host	12.34	0.006	**Bloodmeal**	Mouse	0.886	0.710
Enviro	24.88	<0.001		Prairie Dog	0.874	0.684
* Model Fit: LR χ^2 = 33.01, df = 6, p<0.001, AIC = 80.08				Pig	2.301	0.010
				**Condition**	**Exp (β)[A]**	**p-value**
			**Enviro**	HH	0.824	0.526
				HT	0.241	<0.001
				HH+HT	0.355	0.001

A: Odds ratios (Exp (β)) are reported relative to rat bloodmeal and standard environmental conditions.

Supplemental Figure S1.

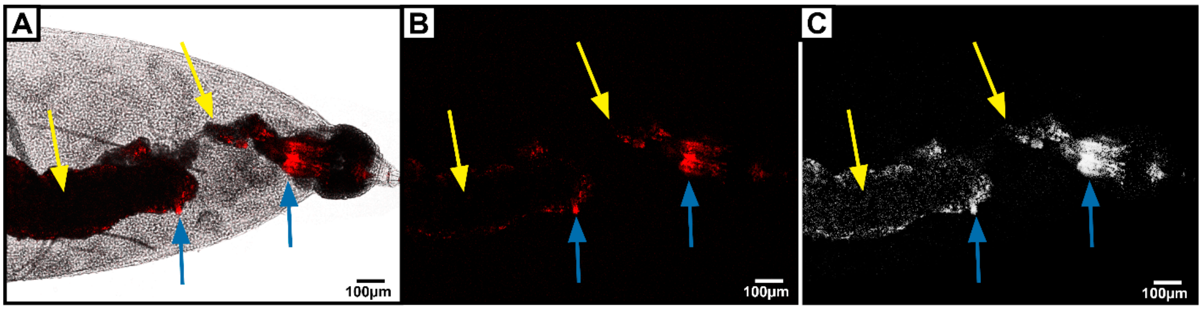

Supplemental Figure S1: *Visualization of proventricular colonization and biofilm formation using confocal microscopy to detect bacteria carrying tdTomato.* (A) RGB image acquired using a Leica SP8 confocal microscope. Fluorescently labeled bacteria appear in red, overlaid on a brightfield image. (B) Red channel only illustrating fluorescent bacteria. (C) RGB is converted to HSB; the Saturation channel from the HSB (hue-saturation-brightness) stack is used to quantify the fluorescent signal by measuring the mean gray value and integrated density in Image J. Yellow arrows indicate areas where the fluorescent signal is quenched and not clearly visible in the red fluorescence channel but is visible in the HSB saturation channel. Blue arrows indicate regions clearly visible in both. The flea shown was infected with pig blood, maintained under standard conditions, and euthanized day 14 post-infection.
