# Supplementary material for: Environmental and Host Blood Interactions Shape Yersinia pestis Dynamics in the Rat Flea, Xenopsylla cheopis
Source: Pathogens. 2026 Jun 16;15(6):639. doi: 10.3390/pathogens15060639 (PMC13305545; doi:10.3390/pathogens15060639)
Supplement: Supplementary file 1 [file pathogens-15-00639-s001.zip › Raw Data.pdf]

Data for crystal violet staining quantification

Raw Absorbance Data								
Environment	Hour	Abs	Environment	Hour	Abs	Environment	Hour	Abs
SRC	24	0.21163	SRC	48	0.16825	SRC	72	0.33588
SRC	24	0.26663	SRC	48	0.14425	SRC	72	0.33788
SRC	24	0.33963	SRC	48	0.06525	SRC	72	0.39588
SRC	24	0.30163	SRC	48	0.10825	SRC	72	0.32988
SRC	24	0.38763	SRC	48	0.07425	SRC	72	0.39488
SRC	24	0.15263	SRC	48	0.08325	SRC	72	0.48888
SRC	24	0.17963	SRC	48	0.09225	SRC	72	0.38988
SRC	24	0.24963	SRC	48	0.11025	SRC	72	0.32088
SRC	24	0.21563	SRC	48	0.15925	SRC	72	0.27988
SRC	24	0.18363	SRC	48	0.14625	SRC	72	0.24388
SRC	24	0.20463	SRC	48	0.19025	SRC	72	0.27888
SRC	24	0.19863	SRC	48	0.19925	SRC	72	0.36988
SRC	24	0.40963	SRC	48	0.11325	SRC	72	0.40288
SRC	24	0.12563	SRC	48	0.18125	SRC	72	0.40588
SRC	24	0.19263	SRC	48	0.16225	SRC	72	0.37888
SRC	24	0.13363	SRC	48	0.17225	SRC	72	0.38688
SRC	24	0.25363	SRC	48	0.12225	SRC	72	0.37288
SRC	24	0.24463	SRC	48	0.13825	SRC	72	0.24088
SRC	24	0.22563	SRC	48	0.07825	SRC	72	0.23688
SRC	24	0.13663	SRC	48	0.10925	SRC	72	0.26388
SRC	24	0.17463	SRC	48	0.08725	SRC	72	0.22788
SRC	24	0.14963	SRC	48	0.09825	SRC	72	0.27288
SRC	24	0.13763	SRC	48	0.08825	SRC	72	0.26588
SRC	24	0.15563	SRC	48	0.05025	SRC	72	0.30388
HT	24	0.35963	HT	48	0.158	HT	72	0.99413
HT	24	0.39563	HT	48	0.195	HT	72	1.63713
HT	24	0.32863	HT	48	0.198	HT	72	0.69513
HT	24	0.35963	HT	48	0.235	HT	72	0.64813
HT	24	0.33663	HT	48	0.19	HT	72	0.91213
HT	24	0.33563	HT	48	0.266	HT	72	0.55713
HT	24	0.40263	HT	48	0.224	HT	72	0.50713
HT	24	0.32963	HT	48	0.193	HT	72	1.02713
HT	24	0.52363	HT	48	0.112	HT	72	0.96613
HT	24	0.33963	HT	48	0.223	HT	72	0.73113
HT	24	0.31763	HT	48	0.257	HT	72	0.72913
HT	24	0.26363	HT	48	0.247	HT	72	0.91913
HT	24	0.25763	HT	48	0.208	HT	72	0.69113
HT	24	0.22263	HT	48	0.233	HT	72	1.06813
HT	24	0.32163	HT	48	0.185	HT	72	0.95513
HT	24	0.39363	HT	48	0.467	HT	72	2.20913
HT	24	0.40763	HT	48	0.22	HT	72	1.44513
HT	24	0.24863	HT	48	0.138	HT	72	0.81313
HT	24	0.30763	HT	48	0.15	HT	72	0.93313
HT	24	0.28163	HT	48	0.151	HT	72	0.97413
HT	24	0.24863	HT	48	0.159	HT	72	1.05413
HT	24	0.30263	HT	48	0.156	HT	72	0.99313
HT	24	0.22063	HT	48	0.181	HT	72	1.32413
HT	24	0.38163	HT	48	0.293	HT	72	1.02713
HH	24	0.41113	HH	48	0.19863	HH	72	0.5265
HH	24	0.32713	HH	48	0.20863	HH	72	0.4845
HH	24	0.29513	HH	48	0.25563	HH	72	0.5835
HH	24	0.35713	HH	48	0.23563	HH	72	0.7085
HH	24	0.34113	HH	48	0.28063	HH	72	0.6975
HH	24	0.26213	HH	48	0.25363	HH	72	0.7225
HH	24	0.28113	HH	48	0.22663	HH	72	0.6885
HH	24	0.32213	HH	48	0.14863	HH	72	0.7325

Average Absorbance		
Environment	Hour	Avg Abs
SRC	24	0.26113
HT	24	0.356
HH	24	0.32463
HH+HT	24	0.27
SRC	24	0.208
HT	24	0.33
HH	24	0.34813
HH+HT	24	0.30213
SRC	24	0.18475
HT	24	0.29988
HH	24	0.335
HH+HT	24	0.315
SRC	48	0.10575
HT	48	0.20738
HH	48	0.226
HH+HT	48	0.15425
SRC	48	0.1655
HT	48	0.2415
HH	48	0.1675
HH+HT	48	0.17213
SRC	48	0.0965
HT	48	0.181
HH	48	0.15175
HH+HT	48	0.14913
SRC	72	0.37425
HT	72	0.87225
HH	72	0.643
HH+HT	72	0.57913
SRC	72	0.34338
HT	72	1.03363
HH	72	0.54888
HH+HT	72	0.45375
SRC	72	0.27313
HT	72	1.0705
HH	72	0.52013
HH+HT	72	0.605

HH	24	0.20013
HH	24	0.30013
HH	24	0.36013
HH	24	0.32513
HH	24	0.29613
HH	24	0.31413
HH	24	0.35013
HH	24	0.63913
HH	24	0.38713
HH	24	0.31813
HH	24	0.32413
HH	24	0.32513
HH	24	0.36213
HH	24	0.34213
HH	24	0.29413
HH	24	0.32713
HH+HT	24	0.36
HH+HT	24	0.307
HH+HT	24	0.214
HH+HT	24	0.2
HH+HT	24	0.249
HH+HT	24	0.257
HH+HT	24	0.277
HH+HT	24	0.296
HH+HT	24	0.293
HH+HT	24	0.317
HH+HT	24	0.306
HH+HT	24	0.301
HH+HT	24	0.304
HH+HT	24	0.273
HH+HT	24	0.29
HH+HT	24	0.333
HH+HT	24	0.327
HH+HT	24	0.314
HH+HT	24	0.307
HH+HT	24	0.294
HH+HT	24	0.298
HH+HT	24	0.286
HH+HT	24	0.314
HH+HT	24	0.38

HH	48	0.15563
HH	48	0.17363
HH	48	0.19363
HH	48	0.16963
HH	48	0.19563
HH	48	0.19163
HH	48	0.13063
HH	48	0.12963
HH	48	0.13263
HH	48	0.14263
HH	48	0.18563
HH	48	0.18563
HH	48	0.14663
HH	48	0.20263
HH	48	0.16263
HH	48	0.05563
HH+HT	48	0.12825
HH+HT	48	0.13625
HH+HT	48	0.17025
HH+HT	48	0.16525
HH+HT	48	0.18325
HH+HT	48	0.17125
HH+HT	48	0.11125
HH+HT	48	0.16825
HH+HT	48	0.13525
HH+HT	48	0.18825
HH+HT	48	0.16225
HH+HT	48	0.23625
HH+HT	48	0.18025
HH+HT	48	0.16225
HH+HT	48	0.14625
HH+HT	48	0.16625
HH+HT	48	0.11225
HH+HT	48	0.14625
HH+HT	48	0.15325
HH+HT	48	0.16825
HH+HT	48	0.17425
HH+HT	48	0.17125
HH+HT	48	0.16725
HH+HT	48	0.10025

HH	72	0.5125
HH	72	0.2875
HH	72	0.4655
HH	72	0.4045
HH	72	0.4775
HH	72	0.6745
HH	72	1.0675
HH	72	0.5015
HH	72	0.4455
HH	72	0.4135
HH	72	0.5315
HH	72	0.3925
HH	72	0.6745
HH	72	0.5825
HH	72	0.5305
HH	72	0.5905
HH+HT	72	0.65475
HH+HT	72	0.59675
HH+HT	72	0.54875
HH+HT	72	0.33575
HH+HT	72	0.62975
HH+HT	72	0.47775
HH+HT	72	0.64875
HH+HT	72	0.74075
HH+HT	72	0.53275
HH+HT	72	0.49675
HH+HT	72	0.49275
HH+HT	72	0.38175
HH+HT	72	0.45075
HH+HT	72	0.48475
HH+HT	72	0.33375
HH+HT	72	0.45675
HH+HT	72	0.57375
HH+HT	72	0.44275
HH+HT	72	0.43175
HH+HT	72	0.48075
HH+HT	72	0.57075
HH+HT	72	0.60975
HH+HT	72	0.70275
HH+HT	72	1.02775

Data for CFU

Host	Day	Enviro	CFUs
Rat	1	SRC	300000000
Rat	1	SRC	9500000
Rat	1	SRC	106000000
Rat	3	SRC	33000000
Rat	3	SRC	5000000
Rat	3	SRC	6700000
Rat	4	SRC	127000
Rat	4	SRC	73000
Rat	4	SRC	98000
Rat	7	SRC	270000
Rat	7	SRC	660000
Rat	7	SRC	320000
Rat	1	HH	200000
Rat	1	HH	6400000000
Rat	1	HH	1980000
Rat	3	HH	2000
Rat	3	HH	0
Rat	3	HH	8000
Rat	4	HH	0
Rat	4	HH	2000
Rat	4	HH	70000
Rat	7	HH	80000
Rat	7	HH	0
Rat	7	HH	5000
Rat	1	HT	38000
Rat	1	HT	53000
Rat	1	HT	0
Rat	3	HT	290000
Rat	3	HT	0
Rat	3	HT	2000
Rat	4	HT	0
Rat	4	HT	60000
Rat	4	HT	7000
Rat	7	HT	2000
Rat	7	HT	96000
Rat	7	HT	136000
Rat	1	HH+HT	5000
Rat	1	HH+HT	2000
Rat	1	HH+HT	3000
Rat	3	HH+HT	0
Rat	3	HH+HT	16000
Rat	3	HH+HT	29000
Rat	4	HH+HT	27000
Rat	4	HH+HT	0
Rat	4	HH+HT	20000
Rat	7	HH+HT	35000
Rat	7	HH+HT	4000
Rat	7	HH+HT	22000

Host	Day	Enviro	CFUs
Mouse	1	SRC	45000
Mouse	1	SRC	3000
Mouse	1	SRC	21000
Mouse	3	SRC	4000
Mouse	3	SRC	10200000
Mouse	3	SRC	12000
Mouse	4	SRC	2000
Mouse	4	SRC	75000
Mouse	4	SRC	96000
Mouse	7	SRC	1340000
Mouse	7	SRC	46000000
Mouse	7	SRC	32000
Mouse	1	HH	0
Mouse	1	HH	0
Mouse	1	HH	119000
Mouse	3	HH	16700000
Mouse	3	HH	46000
Mouse	3	HH	400000
Mouse	4	HH	130000
Mouse	4	HH	0
Mouse	4	HH	3000
Mouse	7	HH	49000
Mouse	7	HH	3000
Mouse	7	HH	109000
Mouse	1	HT	2000
Mouse	1	HT	0
Mouse	1	HT	1000
Mouse	3	HT	0
Mouse	3	HT	11000
Mouse	3	HT	0
Mouse	4	HT	0
Mouse	4	HT	16000
Mouse	4	HT	0
Mouse	7	HT	320000
Mouse	7	HT	9000
Mouse	7	HT	9000
Mouse	1	HH+HT	77000
Mouse	1	HH+HT	0
Mouse	1	HH+HT	0
Mouse	3	HH+HT	170000
Mouse	3	HH+HT	7000
Mouse	3	HH+HT	2000
Mouse	4	HH+HT	490000000
Mouse	4	HH+HT	32000
Mouse	4	HH+HT	0
Mouse	7	HH+HT	10000
Mouse	7	HH+HT	6000
Mouse	7	HH+HT	0

Host	Day	Enviro	CFUs
PDog	1	SRC	1270000
PDog	1	SRC	7300000
PDog	1	SRC	2140000
PDog	3	SRC	5000
PDog	3	SRC	9000
PDog	3	SRC	6000
PDog	4	SRC	0
PDog	4	SRC	48000
PDog	4	SRC	96000
PDog	7	SRC	3000
PDog	7	SRC	0
PDog	7	SRC	10000
PDog	1	HH	49000
PDog	1	HH	149000
PDog	1	HH	88000
PDog	3	HH	170000
PDog	3	HH	0
PDog	3	HH	94000
PDog	4	HH	20000
PDog	4	HH	0
PDog	4	HH	64000
PDog	7	HH	0
PDog	7	HH	47000
PDog	7	HH	26000
PDog	1	HT	43000
PDog	1	HT	53000
PDog	1	HT	30000
PDog	3	HT	0
PDog	3	HT	11000
PDog	3	HT	28000
PDog	4	HT	0
PDog	4	HT	0
PDog	4	HT	0
PDog	7	HT	0
PDog	7	HT	0
PDog	7	HT	0
PDog	1	HH+HT	63000
PDog	1	HH+HT	24000
PDog	1	HH+HT	118000
PDog	3	HH+HT	126000
PDog	3	HH+HT	84000
PDog	3	HH+HT	0
PDog	4	HH+HT	0
PDog	4	HH+HT	162000
PDog	4	HH+HT	49000
PDog	7	HH+HT	28000
PDog	7	HH+HT	39000
PDog	7	HH+HT	32000

Host	Day	Enviro	CFUs
Pig	1	SRC	3600000
Pig	1	SRC	3.4E+07
Pig	1	SRC	1.9E+07
Pig	3	SRC	2.9E+07
Pig	3	SRC	7.2E+07
Pig	3	SRC	2010000
Pig	4	SRC	9.2E+07
Pig	4	SRC	2430000
Pig	4	SRC	3230000
Pig	7	SRC	2910000
Pig	7	SRC	2900000
Pig	7	SRC	2060000
Pig	1	HH	6000
Pig	1	HH	48000
Pig	1	HH	76000
Pig	3	HH	2000
Pig	3	HH	3000
Pig	3	HH	96000
Pig	4	HH	11000
Pig	4	HH	4E+08
Pig	4	HH	4300000
Pig	7	HH	3000
Pig	7	HH	48000
Pig	7	HH	54000
Pig	1	HT	2260000
Pig	1	HT	8.8E+07
Pig	1	HT	7000
Pig	3	HT	250000
Pig	3	HT	1000
Pig	3	HT	200000
Pig	4	HT	0
Pig	4	HT	40000
Pig	4	HT	0
Pig	7	HT	31000
Pig	7	HT	0
Pig	7	HT	43000
Pig	1	HH+HT	5000
Pig	1	HH+HT	2E+07
Pig	1	HH+HT	460000
Pig	3	HH+HT	4000
Pig	3	HH+HT	0
Pig	3	HH+HT	5200000
Pig	4	HH+HT	0
Pig	4	HH+HT	218000
Pig	4	HH+HT	131000
Pig	7	HH+HT	128000
Pig	7	HH+HT	165000
Pig	7	HH+HT	179000

Data for Bloodmeal Digestion Total Protein

Host	Enviro	Infection	Hour	Protein
Rat	SRC	UN	6	120.88
Rat	SRC	UN	6	148.90
Rat	SRC	UN	6	114.14
Rat	HH	UN	6	152.68
Rat	HH	UN	6	196.65
Rat	HH	UN	6	200.14
Rat	HT	UN	6	23.88
Rat	HT	UN	6	24.90
Rat	HT	UN	6	26.34
Rat	HH+HT	UN	6	86.08
Rat	HH+HT	UN	6	121.90
Rat	HH+HT	UN	6	96.74
Rat	SRC	inf	6	50.30
Rat	SRC	inf	6	60.07
Rat	SRC	inf	6	56.45
Rat	HH	inf	6	16.80
Rat	HH	inf	6	30.40
Rat	HH	inf	6	29.62
Rat	HT	inf	6	169.30
Rat	HT	inf	6	164.23
Rat	HT	inf	6	178.28
Rat	HH+HT	inf	6	121.63
Rat	HH+HT	inf	6	160.23
Rat	HH+HT	inf	6	130.28
Mouse	SRC	UN	6	225.80
Mouse	SRC	UN	6	324.12
Mouse	SRC	UN	6	271.86
Mouse	HH	UN	6	184.40
Mouse	HH	UN	6	168.92
Mouse	HH	UN	6	181.86
Mouse	HT	UN	6	186.80
Mouse	HT	UN	6	140.92
Mouse	HT	UN	6	193.86
Mouse	HH+HT	UN	6	152.40
Mouse	HH+HT	UN	6	128.72
Mouse	HH+HT	UN	6	199.86
Mouse	SRC	inf	6	77.36
Mouse	SRC	inf	6	71.52
Mouse	SRC	inf	6	52.64
Mouse	HH	inf	6	111.16
Mouse	HH	inf	6	101.92
Mouse	HH	inf	6	125.04
Mouse	HT	inf	6	241.76
Mouse	HT	inf	6	242.32
Mouse	HT	inf	6	253.44
Mouse	HH+HT	inf	6	192.96
Mouse	HH+HT	inf	6	187.72
Mouse	HH+HT	inf	6	207.24
PDog	SRC	UN	6	96.56
PDog	SRC	UN	6	82.38
PDog	SRC	UN	6	108.47
PDog	HH	UN	6	498.16
PDog	HH	UN	6	402.18
PDog	HH	UN	6	324.80
PDog	HT	UN	6	334.56
PDog	HT	UN	6	328.78
PDog	HT	UN	6	303.63
PDog	HH+HT	UN	6	266.16
PDog	HH+HT	UN	6	242.18
PDog	HH+HT	UN	6	219.47
PDog	SRC	inf	6	277.72
PDog	SRC	inf	6	180.97
PDog	SRC	inf	6	178.02
PDog	HH	inf	6	212.32
PDog	HH	inf	6	184.80
PDog	HH	inf	6	194.52
PDog	HT	inf	6	105.32
PDog	HT	inf	6	120.13
PDog	HT	inf	6	72.85
PDog	HH+HT	inf	6	180.72
PDog	HH+HT	inf	6	123.97
PDog	HH+HT	inf	6	183.02
Pig	SRC	UN	6	172.75
Pig	SRC	UN	6	210.25
Pig	SRC	UN	6	186.80
Pig	HH	UN	6	244.75
Pig	HH	UN	6	239.50
Pig	HH	UN	6	243.30
Pig	HT	UN	6	96.50
Pig	HT	UN	6	110.50
Pig	HT	UN	6	129.05
Pig	HH+HT	UN	6	162.50
Pig	HH+HT	UN	6	151.25
Pig	HH+HT	UN	6	166.30
Pig	SRC	inf	6	162.22
Pig	SRC	inf	6	226.60
Pig	SRC	inf	6	174.08
Pig	HH	inf	6	52.42
Pig	HH	inf	6	72.10
Pig	HH	inf	6	59.08
Pig	HT	inf	6	40.02
Pig	HT	inf	6	44.35
Pig	HT	inf	6	43.83
Pig	HH+HT	inf	6	159.22
Pig	HH+HT	inf	6	175.60
Pig	HH+HT	inf	6	188.58

Host	Enviro	Infection	Hour	Protein
Rat	SRC	UN	12	66.88
Rat	SRC	UN	12	79.65
Rat	SRC	UN	12	52.14
Rat	HH	UN	12	51.88
Rat	HH	UN	12	127.15
Rat	HH	UN	12	94.34
Rat	HT	UN	12	71.48
Rat	HT	UN	12	95.15
Rat	HT	UN	12	82.54
Rat	HH+HT	UN	12	44.88
Rat	HH+HT	UN	12	119.9
Rat	HH+HT	UN	12	87.74
Rat	SRC	INF	12	83.47
Rat	SRC	INF	12	86.57
Rat	SRC	INF	12	115.45
Rat	HH	INF	12	32.63
Rat	HH	INF	12	35.4
Rat	HH	INF	12	53.12
Rat	HT	INF	12	74.47
Rat	HT	INF	12	51.73
Rat	HT	INF	12	62.28
Rat	HH+HT	INF	12	63.3
Rat	HH+HT	INF	12	48.9
Rat	HH+HT	INF	12	81.12
Mouse	SRC	UN	12	221
Mouse	SRC	UN	12	164.52
Mouse	SRC	UN	12	191.86
Mouse	HH	UN	12	108.4
Mouse	HH	UN	12	116.72
Mouse	HH	UN	12	105.86
Mouse	HT	UN	12	116.8
Mouse	HT	UN	12	121.12
Mouse	HT	UN	12	139.06
Mouse	HH+HT	UN	12	126.6
Mouse	HH+HT	UN	12	143.52
Mouse	HH+HT	UN	12	220.86
Mouse	SRC	INF	12	140.76
Mouse	SRC	INF	12	136.12
Mouse	SRC	INF	12	144.24
Mouse	HH	INF	12	96.16
Mouse	HH	INF	12	92.12
Mouse	HH	INF	12	128.64
Mouse	HT	INF	12	127.56
Mouse	HT	INF	12	127.12
Mouse	HT	INF	12	146.84
Mouse	HH+HT	INF	12	149.76
Mouse	HH+HT	INF	12	223.72
Mouse	HH+HT	INF	12	178.24
PDog	SRC	UN	12	91.36
PDog	SRC	UN	12	88.18
PDog	SRC	UN	12	111.3
PDog	HH	UN	12	329.16
PDog	HH	UN	12	263.38
PDog	HH	UN	12	272.13
PDog	HT	UN	12	150.76
PDog	HT	UN	12	127.98
PDog	HT	UN	12	130.13
PDog	HH+HT	UN	12	57.76
PDog	HH+HT	UN	12	69.18
PDog	HH+HT	UN	12	46.47
PDog	SRC	INF	12	43.72
PDog	SRC	INF	12	39.8
PDog	SRC	INF	12	31.85
PDog	HH	INF	12	192.92
PDog	HH	INF	12	166.13
PDog	HH	INF	12	108.35
PDog	HT	INF	12	86.32
PDog	HT	INF	12	64.47
PDog	HT	INF	12	88.02
PDog	HH+HT	INF	12	130.92
PDog	HH+HT	INF	12	95.63
PDog	HH+HT	INF	12	127.52
Pig	SRC	UN	12	164.5
Pig	SRC	UN	12	204.25
Pig	SRC	UN	12	170.55
Pig	HH	UN	12	201.5
Pig	HH	UN	12	194
Pig	HH	UN	12	188.05
Pig	HT	UN	12	197.25
Pig	HT	UN	12	161
Pig	HT	UN	12	173.3
Pig	HH+HT	UN	12	235.25
Pig	HH+HT	UN	12	239.25
Pig	HH+HT	UN	12	241.05
Pig	SRC	INF	12	185.02
Pig	SRC	INF	12	155.85
Pig	SRC	INF	12	158.83
Pig	HH	INF	12	114.82
Pig	HH	INF	12	112.35
Pig	HH	INF	12	129.33
Pig	HT	INF	12	195.62
Pig	HT	INF	12	183.6
Pig	HT	INF	12	191.33
Pig	HH+HT	INF	12	27.62
Pig	HH+HT	INF	12	38.6
Pig	HH+HT	INF	12	40.83

Host	Enviro	Infection	Hour	Protein
Rat	SRC	UN	18	94.28
Rat	SRC	UN	18	157.9
Rat	SRC	UN	18	158.94
Rat	HH	UN	18	114.28
Rat	HH	UN	18	154.9
Rat	HH	UN	18	158.34
Rat	HT	UN	18	51.68
Rat	HT	UN	18	63.65
Rat	HT	UN	18	58.94
Rat	HH+HT	UN	18	91.88
Rat	HH+HT	UN	18	120.65
Rat	HH+HT	UN	18	89.94
Rat	SRC	inf	18	53.13
Rat	SRC	inf	18	46.73
Rat	SRC	inf	18	46.12
Rat	HH	inf	18	85.97
Rat	HH	inf	18	85.57
Rat	HH	inf	18	91.62
Rat	HT	inf	18	120.47
Rat	HT	inf	18	139.57
Rat	HT	inf	18	104.45
Rat	HH+HT	inf	18	53.13
Rat	HH+HT	inf	18	38.57
Rat	HH+HT	inf	18	34.62
Mouse	SRC	UN	18	87.8
Mouse	SRC	UN	18	90.92
Mouse	SRC	UN	18	93.86
Mouse	HH	UN	18	158.6
Mouse	HH	UN	18	173.52
Mouse	HH	UN	18	241.66
Mouse	HT	UN	18	112.8
Mouse	HT	UN	18	102.52
Mouse	HT	UN	18	114.26
Mouse	HH+HT	UN	18	149.6
Mouse	HH+HT	UN	18	161.12
Mouse	HH+HT	UN	18	143.06
Mouse	SRC	inf	18	78.56
Mouse	SRC	inf	18	33.12
Mouse	SRC	inf	18	76.44
Mouse	HH	inf	18	94.16
Mouse	HH	inf	18	99.72
Mouse	HH	inf	18	114.44
Mouse	HT	inf	18	50.96
Mouse	HT	inf	18	68.12
Mouse	HT	inf	18	68.44
Mouse	HH+HT	inf	18	112.16
Mouse	HH+HT	inf	18	104.72
Mouse	HH+HT	inf	18	118.44
PDog	SRC	UN	18	258.56
PDog	SRC	UN	18	228.38
PDog	SRC	UN	18	221.47
PDog	HH	UN	18	212.56
PDog	HH	UN	18	177.78
PDog	HH	UN	18	153.47
PDog	HT	UN	18	145.56
PDog	HT	UN	18	191.98
PDog	HT	UN	18	135.63
PDog	HH+HT	UN	18	184.16
PDog	HH+HT	UN	18	160.58
PDog	HH+HT	UN	18	151.97
PDog	SRC	inf	18	50.92
PDog	SRC	inf	18	45.47
PDog	SRC	inf	18	43.68
PDog	HH	inf	18	95.92
PDog	HH	inf	18	97.47
PDog	HH	inf	18	59.52
PDog	HT	inf	18	133.52
PDog	HT	inf	18	125.13
PDog	HT	inf	18	110.02
PDog	HH+HT	inf	18	71.92
PDog	HH+HT	inf	18	67.8
PDog	HH+HT	inf	18	90.18
Pig	SRC	UN	18	66.25
Pig	SRC	UN	18	74.75
Pig	SRC	UN	18	74.55
Pig	HH	UN	18	40.75
Pig	HH	UN	18	43.25
Pig	HH	UN	18	92.55
Pig	HT	UN	18	150.5
Pig	HT	UN	18	149
Pig	HT	UN	18	168.8
Pig	HH+HT	UN	18	400.75
Pig	HH+HT	UN	18	371.5
Pig	HH+HT	UN	18	426.05
Pig	SRC	inf	18	33.82
Pig	SRC	inf	18	41.6
Pig	SRC	inf	18	26.58
Pig	HH	inf	18	234.22
Pig	HH	inf	18	260.1
Pig	HH	inf	18	307.08
Pig	HT	inf	18	78.42
Pig	HT	inf	18	93.6
Pig	HT	inf	18	93.33
Pig	HH+HT	inf	18	18.62
Pig	HH+HT	inf	18	13.6
Pig	HH+HT	inf	18	13.08

Data for Flea Mortality

Species	RH	Temp	Time P.I.	Starting sample size	# Analyzed confocal	# damaged during processing	Total Confocal	# Dead	# survived	% survival	% mortality
Rat	70	25	7	19	10	1	11	8	11	0.57895	0.42105263
Rat	70	30	7	32	12	0	12	20	12	0.375	0.625
Rat	80	25	7	23	12	1	13	10	13	0.56522	0.43478261
Rat	80	30	7	21	10	0	10	11	10	0.47619	0.52380952
Mouse	70	25	7	27	11	2	13	14	13	0.48148	0.51851852
Mouse	70	30	7	26	10	0	10	16	10	0.38462	0.61538462
Mouse	80	25	7	22	10	0	10	12	10	0.45455	0.54545455
Mouse	80	30	7	22	9	0	9	13	9	0.40909	0.59090909
PDog	70	25	7	19	12	0	12	7	12	0.63158	0.36842105
PDog	70	30	7	21	12	1	13	8	13	0.61905	0.38095238
PDog	80	25	7	19	10	1	11	8	11	0.57895	0.42105263
PDog	80	30	7	24	9	0	9	15	9	0.375	0.625
Pig	70	25	7	15	10	0	10	5	10	0.66667	0.33333333
Pig	70	30	7	16	11	0	11	5	11	0.6875	0.3125
Pig	80	25	7	16	10	1	11	5	11	0.6875	0.3125
Pig	80	30	7	14	9	0	9	5	9	0.64286	0.35714286
Rat	70	25	14	25	12	1	13	12	13	0.52	0.48
Rat	70	30	14	20	3	0	3	17	3	0.15	0.85
Rat	80	25	14	22	10	0	10	12	10	0.45455	0.54545455
Rat	80	30	14	24	5	0	5	19	5	0.20833	0.79166667
Mouse	70	25	14	24	9	0	9	15	9	0.375	0.625
Mouse	70	30	14	10	1	0	1	9	1	0.1	0.9
Mouse	80	25	14	27	10	0	10	17	10	0.37037	0.62962963
Mouse	80	30	14	30	10	0	10	20	10	0.33333	0.66666667
PDog	70	25	14	18	9	1	10	8	10	0.55556	0.44444444
PDog	70	30	14	20	2	0	2	18	2	0.1	0.9
PDog	80	25	14	25	13	0	13	12	13	0.52	0.48
PDog	80	30	14	26	2	0	2	24	2	0.07692	0.92307692
Pig	70	25	14	18	11	0	11	7	11	0.61111	0.38888889
Pig	70	30	14	34	14	0	14	20	14	0.41176	0.58823529
Pig	80	25	14	20	9	1	10	10	10	0.5	0.5
Pig	80	30	14	20	10	0	10	10	10	0.5	0.5

Data for RFUs

Host	Enviro	Day	Prov_RFU
Rat	SRC	1	56.42
Rat	SRC	1	29.19
Rat	SRC	1	57.88
Rat	SRC	1	96.99
Rat	SRC	1	22.74
Rat	SRC	1	9.54
Rat	SRC	1	30.34
Rat	SRC	1	79.31
Rat	SRC	1	33.55
Rat	SRC	1	94.88
Rat	HH	1	76.94
Rat	HH	1	43.08
Rat	HH	1	44.75
Rat	HH	1	34.96
Rat	HH	1	25.07
Rat	HH	1	32.09
Rat	HH	1	39.35
Rat	HH	1	41.02
Rat	HH	1	46.94
Rat	HH	1	44.91
Rat	HT	1	66.60
Rat	HT	1	109.71
Rat	HT	1	93.83
Rat	HT	1	79.92
Rat	HT	1	56.05
Rat	HT	1	72.92
Rat	HT	1	76.48
Rat	HT	1	101.73
Rat	HT	1	34.44
Rat	HT	1	10.12
Rat	HH+HT	1	6.46
Rat	HH+HT	1	5.85
Rat	HH+HT	1	0.00
Rat	HH+HT	1	8.18
Rat	HH+HT	1	0.00
Rat	HH+HT	1	0.00
Rat	HH+HT	1	1.68
Rat	HH+HT	1	4.41
Rat	HH+HT	1	0.00
Rat	HH+HT	1	6.54
Mouse	SRC	1	64.14
Mouse	SRC	1	21.20
Mouse	SRC	1	102.72
Mouse	SRC	1	41.66
Mouse	SRC	1	9.45
Mouse	SRC	1	15.61
Mouse	SRC	1	3.53
Mouse	SRC	1	7.47
Mouse	SRC	1	18.56
Mouse	SRC	1	121.19
Mouse	HH	1	148.60
Mouse	HH	1	100.32
Mouse	HH	1	85.69
Mouse	HH	1	75.17
Mouse	HH	1	1.97
Mouse	HH	1	5.13
Mouse	HH	1	6.44
Mouse	HH	1	127.73
Mouse	HH	1	2.61
Mouse	HH	1	29.19
Mouse	HT	1	11.61
Mouse	HT	1	20.18
Mouse	HT	1	12.06
Mouse	HT	1	19.81
Mouse	HT	1	43.33
Mouse	HT	1	20.42
Mouse	HT	1	0.00
Mouse	HT	1	5.67
Mouse	HT	1	1.04
Mouse	HT	1	1.75
Mouse	HH+HT	1	80.66
Mouse	HH+HT	1	73.25
Mouse	HH+HT	1	37.74
Mouse	HH+HT	1	139.61
Mouse	HH+HT	1	99.73
Mouse	HH+HT	1	131.04
Mouse	HH+HT	1	116.07
Mouse	HH+HT	1	49.35
Mouse	HH+HT	1	70.96
Mouse	HH+HT	1	108.15

Host	Enviro	Day	Prov_RFU
Rat	SRC	3	28.99
Rat	SRC	3	163.46
Rat	SRC	3	37.96
Rat	SRC	3	87.38
Rat	SRC	3	145.68
Rat	SRC	3	177.67
Rat	SRC	3	210.67
Rat	SRC	3	334.42
Rat	SRC	3	14.00
Rat	SRC	3	84.08
Rat	HH	3	134.99
Rat	HH	3	9.50
Rat	HH	3	65.56
Rat	HH	3	130.18
Rat	HH	3	21.82
Rat	HH	3	23.60
Rat	HH	3	24.38
Rat	HH	3	1.64
Rat	HH	3	7.54
Rat	HH	3	34.14
Rat	HT	3	67.31
Rat	HT	3	65.84
Rat	HT	3	73.90
Rat	HT	3	104.72
Rat	HT	3	49.13
Rat	HT	3	77.22
Rat	HT	3	92.08
Rat	HT	3	76.68
Rat	HT	3	49.42
Rat	HT	3	5.60
Rat	HH+HT	3	0.00
Rat	HH+HT	3	46.43
Rat	HH+HT	3	25.30
Rat	HH+HT	3	6.31
Rat	HH+HT	3	0.79
Rat	HH+HT	3	10.04
Rat	HH+HT	3	5.72
Rat	HH+HT	3	0.00
Rat	HH+HT	3	9.87
Rat	HH+HT	3	2.56
Mouse	SRC	3	6.68
Mouse	SRC	3	41.52
Mouse	SRC	3	49.23
Mouse	SRC	3	175.77
Mouse	SRC	3	56.28
Mouse	SRC	3	75.05
Mouse	SRC	3	193.65
Mouse	SRC	3	15.80
Mouse	SRC	3	28.08
Mouse	SRC	3	30.53
Mouse	HH	3	15.85
Mouse	HH	3	28.79
Mouse	HH	3	36.75
Mouse	HH	3	117.79
Mouse	HH	3	0.00
Mouse	HH	3	0.00
Mouse	HH	3	0.00
Mouse	HH	3	15.58
Mouse	HH	3	0.08
Mouse	HH	3	0.00
Mouse	HT	3	40.77
Mouse	HT	3	0.00
Mouse	HT	3	3.16
Mouse	HT	3	0.00
Mouse	HT	3	4.13
Mouse	HT	3	1.27
Mouse	HT	3	13.73
Mouse	HT	3	2.13
Mouse	HT	3	17.59
Mouse	HT	3	41.81
Mouse	HH+HT	3	67.24
Mouse	HH+HT	3	0.00
Mouse	HH+HT	3	56.24
Mouse	HH+HT	3	2.58
Mouse	HH+HT	3	14.36
Mouse	HH+HT	3	45.98
Mouse	HH+HT	3	98.58
Mouse	HH+HT	3	1.62
Mouse	HH+HT	3	3.24
Mouse	HH+HT	3	0.00

Host	Enviro	Day	Prov_RFU
Rat	SRC	4	160.97
Rat	SRC	4	27.01
Rat	SRC	4	80.68
Rat	SRC	4	96.82
Rat	SRC	4	84.96
Rat	SRC	4	58.36
Rat	SRC	4	144.85
Rat	SRC	4	129.48
Rat	SRC	4	165.44
Rat	SRC	4	64.39
Rat	HH	4	103.97
Rat	HH	4	88.49
Rat	HH	4	28.52
Rat	HH	4	49.32
Rat	HH	4	42.24
Rat	HH	4	18.85
Rat	HH	4	17.99
Rat	HH	4	271.88
Rat	HH	4	33.20
Rat	HH	4	21.61
Rat	HT	4	35.96
Rat	HT	4	34.36
Rat	HT	4	13.59
Rat	HT	4	35.36
Rat	HT	4	22.25
Rat	HT	4	42.63
Rat	HT	4	38.53
Rat	HT	4	21.52
Rat	HT	4	26.59
Rat	HT	4	18.39
Rat	HH+HT	4	113.27
Rat	HH+HT	4	19.60
Rat	HH+HT	4	77.93
Rat	HH+HT	4	81.13
Rat	HH+HT	4	90.73
Rat	HH+HT	4	33.02
Rat	HH+HT	4	51.44
Rat	HH+HT	4	60.67
Rat	HH+HT	4	40.85
Rat	HH+HT	4	241.62
Mouse	SRC	4	21.02
Mouse	SRC	4	30.50
Mouse	SRC	4	26.20
Mouse	SRC	4	91.74
Mouse	SRC	4	36.26
Mouse	SRC	4	22.08
Mouse	SRC	4	17.53
Mouse	SRC	4	39.49
Mouse	SRC	4	182.00
Mouse	SRC	4	179.14
Mouse	HH	4	281.36
Mouse	HH	4	34.72
Mouse	HH	4	87.82
Mouse	HH	4	0.00
Mouse	HH	4	0.00
Mouse	HH	4	0.00
Mouse	HH	4	0.00
Mouse	HH	4	0.00
Mouse	HH	4	0.00
Mouse	HH	4	17.64
Mouse	HT	4	49.19
Mouse	HT	4	23.66
Mouse	HT	4	96.97
Mouse	HT	4	59.47
Mouse	HT	4	0.96
Mouse	HT	4	5.75
Mouse	HT	4	0.00
Mouse	HT	4	0.00
Mouse	HT	4	13.74
Mouse	HT	4	0.71
Mouse	HH+HT	4	10.52
Mouse	HH+HT	4	47.25
Mouse	HH+HT	4	13.35
Mouse	HH+HT	4	80.06
Mouse	HH+HT	4	58.58
Mouse	HH+HT	4	38.54
Mouse	HH+HT	4	10.47
Mouse	HH+HT	4	7.98
Mouse	HH+HT	4	0.00
Mouse	HH+HT	4	24.73

Host	Enviro	Day	Prov_RFU	Overall_RFU
Rat	SRC	7	111.71	488.30
Rat	SRC	7	41.51	691.01
Rat	SRC	7	19.79	675.25
Rat	SRC	7	21.84	163.73
Rat	SRC	7	128.84	780.37
Rat	SRC	7	68.88	546.40
Rat	SRC	7	128.38	858.78
Rat	SRC	7	26.03	269.47
Rat	SRC	7	41.45	484.39
Rat	SRC	7	57.48	454.52
Rat	HH	7	238.03	3916.71
Rat	HH	7	87.53	575.63
Rat	HH	7	16.86	83.27
Rat	HH	7	33.51	250.20
Rat	HH	7	53.70	203.45
Rat	HH	7	67.38	1729.88
Rat	HH	7	75.73	1033.16
Rat	HH	7	87.87	2181.00
Rat	HH	7	62.63	959.68
Rat	HH	7	95.30	375.07
Rat	HT	7	81.91	1123.74
Rat	HT	7	58.41	223.64
Rat	HT	7	75.85	264.53
Rat	HT	7	51.88	598.91
Rat	HT	7	24.42	179.22
Rat	HT	7	48.56	199.17
Rat	HT	7	82.28	725.05
Rat	HT	7	13.59	66.55
Rat	HT	7	5.89	0.00
Rat	HT	7	32.26	436.11
Rat	HH+HT	7	67.06	483.67
Rat	HH+HT	7	39.55	249.47
Rat	HH+HT	7	113.59	918.60
Rat	HH+HT	7	54.38	288.39
Rat	HH+HT	7	62.40	495.02
Rat	HH+HT	7	89.73	676.13
Rat	HH+HT	7	70.71	607.68
Rat	HH+HT	7	4.89	0.00
Rat	HH+HT	7	28.41	34.23
Rat	HH+HT	7	3.50	230.87
Mouse	SRC	7	166.64	1601.66
Mouse	SRC	7	25.41	344.04
Mouse	SRC	7	62.37	378.01
Mouse	SRC	7	3.90	99.83
Mouse	SRC	7	55.27	963.51
Mouse	SRC	7	11.57	47.75
Mouse	SRC	7	20.85	96.16
Mouse	SRC	7	69.15	589.98
Mouse	SRC	7	4.73	0.00
Mouse	SRC	7	193.50	672.21
Mouse	HH	7	0.00	0.00
Mouse	HH	7	0.00	0.00
Mouse	HH	7	0.00	0.00
Mouse	HH	7	0.00	0.00
Mouse	HH	7	0.00	0.00
Mouse	HH	7	2.49	0.00
Mouse	HH	7	0.00	0.00
Mouse	HH	7	2.05	43.79
Mouse	HH	7	78.46	435.16
Mouse	HH	7	7.50	0.00
Mouse	HT	7	147.82	2553.13
Mouse	HT	7	9.96	5.40
Mouse	HT	7	42.01	241.67
Mouse	HT	7	1.37	0.00
Mouse	HT	7	0.00	0.00
Mouse	HT	7	0.00	0.00
Mouse	HT	7	23.37	53.74
Mouse	HT	7	0.00	0.00
Mouse	HT	7	1.66	0.00
Mouse	HT	7	2.81	0.00
Mouse	HH+HT	7	0.33	0.00
Mouse	HH+HT	7	1.09	0.00
Mouse	HH+HT	7	0.59	0.00
Mouse	HH+HT	7	113.65	320.65
Mouse	HH+HT	7	27.01	101.16
Mouse	HH+HT	7	0.00	0.00
Mouse	HH+HT	7	5.19	0.00
Mouse	HH+HT	7	0.00	0.00
Mouse	HH+HT	7	2.86	0.00
Mouse	HH+HT	7	No Sample	

PDog	SRC	1	42.89
PDog	SRC	1	27.93
PDog	SRC	1	28.69
PDog	SRC	1	21.04
PDog	SRC	1	60.92
PDog	SRC	1	16.54
PDog	SRC	1	0.00
PDog	SRC	1	18.03
PDog	SRC	1	0.00
PDog	SRC	1	0.00
PDog	HH	1	136.21
PDog	HH	1	44.95
PDog	HH	1	69.94
PDog	HH	1	92.18
PDog	HH	1	123.86
PDog	HH	1	71.83
PDog	HH	1	34.77
PDog	HH	1	8.03
PDog	HH	1	1.59
PDog	HH	1	0.27
PDog	HT	1	67.30
PDog	HT	1	26.01
PDog	HT	1	35.51
PDog	HT	1	34.42
PDog	HT	1	61.49
PDog	HT	1	134.31
PDog	HT	1	100.22
PDog	HT	1	41.07
PDog	HT	1	151.93
PDog	HT	1	193.33
PDog	HH+HT	1	14.42
PDog	HH+HT	1	29.23
PDog	HH+HT	1	22.40
PDog	HH+HT	1	36.45
PDog	HH+HT	1	6.59
PDog	HH+HT	1	97.69
PDog	HH+HT	1	0.00
PDog	HH+HT	1	51.74
PDog	HH+HT	1	0.00
PDog	HH+HT	1	0.83
Pig	SRC	1	158.11
Pig	SRC	1	68.77
Pig	SRC	1	9.88
Pig	SRC	1	20.49
Pig	SRC	1	9.57
Pig	SRC	1	30.32
Pig	SRC	1	13.16
Pig	SRC	1	134.67
Pig	SRC	1	0.00
Pig	SRC	1	5.61
Pig	HH	1	34.00
Pig	HH	1	48.72
Pig	HH	1	14.72
Pig	HH	1	14.48
Pig	HH	1	35.95
Pig	HH	1	5.73
Pig	HH	1	0.85
Pig	HH	1	35.42
Pig	HH	1	8.47
Pig	HH	1	200.58
Pig	HT	1	33.95
Pig	HT	1	39.69
Pig	HT	1	31.15
Pig	HT	1	57.89
Pig	HT	1	113.64
Pig	HT	1	112.84
Pig	HT	1	49.07
Pig	HT	1	83.17
Pig	HT	1	30.25
Pig	HT	1	70.82
Pig	HH+HT	1	136.20
Pig	HH+HT	1	72.63
Pig	HH+HT	1	118.81
Pig	HH+HT	1	183.33
Pig	HH+HT	1	79.78
Pig	HH+HT	1	53.19
Pig	HH+HT	1	70.72
Pig	HH+HT	1	516.58
Pig	HH+HT	1	65.58
Pig	HH+HT	1	99.44

PDog	SRC	3	11.10
PDog	SRC	3	26.85
PDog	SRC	3	82.79
PDog	SRC	3	0.00
PDog	SRC	3	0.00
PDog	SRC	3	0.01
PDog	SRC	3	0.00
PDog	SRC	3	1.01
PDog	SRC	3	No Sample
PDog	SRC	3	No Sample
PDog	HH	3	4.12
PDog	HH	3	17.59
PDog	HH	3	0.00
PDog	HH	3	59.04
PDog	HH	3	216.10
PDog	HH	3	17.25
PDog	HH	3	6.74
PDog	HH	3	26.90
PDog	HH	3	5.61
PDog	HH	3	0.00
PDog	HT	3	24.29
PDog	HT	3	14.58
PDog	HT	3	36.50
PDog	HT	3	10.22
PDog	HT	3	38.89
PDog	HT	3	34.71
PDog	HT	3	3.43
PDog	HT	3	115.54
PDog	HT	3	4.36
PDog	HT	3	0.00
PDog	HH+HT	3	39.70
PDog	HH+HT	3	46.08
PDog	HH+HT	3	3.73
PDog	HH+HT	3	15.88
PDog	HH+HT	3	6.46
PDog	HH+HT	3	45.89
PDog	HH+HT	3	0.38
PDog	HH+HT	3	No Sample
PDog	HH+HT	3	No Sample
PDog	HH+HT	3	No Sample
Pig	SRC	3	129.25
Pig	SRC	3	14.16
Pig	SRC	3	33.12
Pig	SRC	3	0.00
Pig	SRC	3	53.81
Pig	SRC	3	8.22
Pig	SRC	3	286.47
Pig	SRC	3	129.80
Pig	SRC	3	79.61
Pig	SRC	3	112.59
Pig	HH	3	99.96
Pig	HH	3	246.03
Pig	HH	3	151.93
Pig	HH	3	87.73
Pig	HH	3	145.83
Pig	HH	3	174.95
Pig	HH	3	92.57
Pig	HH	3	96.86
Pig	HH	3	3.66
Pig	HH	3	2.34
Pig	HT	3	78.43
Pig	HT	3	54.37
Pig	HT	3	25.69
Pig	HT	3	27.06
Pig	HT	3	242.77
Pig	HT	3	6.29
Pig	HT	3	136.54
Pig	HT	3	120.41
Pig	HT	3	62.98
Pig	HT	3	162.68
Pig	HH+HT	3	48.23
Pig	HH+HT	3	107.38
Pig	HH+HT	3	175.94
Pig	HH+HT	3	42.39
Pig	HH+HT	3	38.33
Pig	HH+HT	3	81.55
Pig	HH+HT	3	109.45
Pig	HH+HT	3	93.45
Pig	HH+HT	3	131.08
Pig	HH+HT	3	239.70

PDog	SRC	4	10.30
PDog	SRC	4	55.53
PDog	SRC	4	29.87
PDog	SRC	4	22.19
PDog	SRC	4	27.84
PDog	SRC	4	0.50
PDog	SRC	4	12.52
PDog	SRC	4	0.00
PDog	SRC	4	1.81
PDog	SRC	4	No Sample
PDog	HH	4	50.13
PDog	HH	4	30.81
PDog	HH	4	54.91
PDog	HH	4	88.48
PDog	HH	4	5.77
PDog	HH	4	131.77
PDog	HH	4	6.98
PDog	HH	4	3.43
PDog	HH	4	0.16
PDog	HH	4	0.00
PDog	HT	4	23.87
PDog	HT	4	48.00
PDog	HT	4	4.60
PDog	HT	4	25.47
PDog	HT	4	59.90
PDog	HT	4	63.15
PDog	HT	4	81.63
PDog	HT	4	57.39
PDog	HT	4	80.09
PDog	HT	4	28.67
PDog	HH+HT	4	19.69
PDog	HH+HT	4	15.38
PDog	HH+HT	4	38.37
PDog	HH+HT	4	16.12
PDog	HH+HT	4	9.05
PDog	HH+HT	4	3.42
PDog	HH+HT	4	0.05
PDog	HH+HT	4	0.00
PDog	HH+HT	4	2.80
PDog	HH+HT	4	No Sample
Pig	SRC	4	699.05
Pig	SRC	4	200.94
Pig	SRC	4	57.66
Pig	SRC	4	35.85
Pig	SRC	4	122.62
Pig	SRC	4	21.35
Pig	SRC	4	9.51
Pig	SRC	4	79.06
Pig	SRC	4	29.70
Pig	SRC	4	34.52
Pig	HH	4	47.55
Pig	HH	4	16.88
Pig	HH	4	9.84
Pig	HH	4	45.98
Pig	HH	4	41.12
Pig	HH	4	242.69
Pig	HH	4	7.32
Pig	HH	4	0.00
Pig	HH	4	2.10
Pig	HH	4	12.00
Pig	HT	4	48.08
Pig	HT	4	12.17
Pig	HT	4	74.73
Pig	HT	4	83.59
Pig	HT	4	169.16
Pig	HT	4	147.13
Pig	HT	4	76.43
Pig	HT	4	34.01
Pig	HT	4	293.02
Pig	HT	4	66.09
Pig	HH+HT	4	132.12
Pig	HH+HT	4	105.45
Pig	HH+HT	4	0.00
Pig	HH+HT	4	27.37
Pig	HH+HT	4	75.79
Pig	HH+HT	4	239.17
Pig	HH+HT	4	209.98
Pig	HH+HT	4	181.22
Pig	HH+HT	4	407.03
Pig	HH+HT	4	371.69

PDog	SRC	7	14.77	450.86
PDog	SRC	7	18.92	377.67
PDog	SRC	7	8.30	135.20
PDog	SRC	7	87.47	394.82
PDog	SRC	7	91.89	1103.53
PDog	SRC	7	47.38	516.33
PDog	SRC	7	34.17	590.98
PDog	SRC	7	46.58	735.13
PDog	SRC	7	55.75	367.88
PDog	SRC	7	26.30	106.06
PDog	HH	7	90.00	947.89
PDog	HH	7	27.04	196.88
PDog	HH	7	461.77	1245.61
PDog	HH	7	164.26	2275.78
PDog	HH	7	175.81	1041.92
PDog	HH	7	221.26	1259.99
PDog	HH	7	142.79	687.81
PDog	HH	7	306.66	1341.34
PDog	HH	7	0.95	0.00
PDog	HH	7	24.41	305.57
PDog	HT	7	14.03	179.20
PDog	HT	7	24.04	392.99
PDog	HT	7	30.80	391.98
PDog	HT	7	10.78	104.16
PDog	HT	7	0.00	33.19
PDog	HT	7	0.00	0.00
PDog	HT	7	31.27	40.32
PDog	HT	7	35.65	209.86
PDog	HT	7	25.52	228.07
PDog	HT	7	20.56	90.14
PDog	HH+HT	7	163.57	3243.45
PDog	HH+HT	7	13.87	107.44
PDog	HH+HT	7	19.31	713.69
PDog	HH+HT	7	58.68	327.69
PDog	HH+HT	7	25.39	436.45
PDog	HH+HT	7	14.66	46.80
PDog	HH+HT	7	13.06	271.08
PDog	HH+HT	7	10.18	0.00
PDog	HH+HT	7	0.00	0.00
PDog	HH+HT	7	No Sample	
Pig	SRC	7	27.97	433.53
Pig	SRC	7	98.96	1112.56
Pig	SRC	7	55.32	384.86
Pig	SRC	7	29.15	431.05
Pig	SRC	7	352.52	3204.80
Pig	SRC	7	36.37	1295.67
Pig	SRC	7	59.42	1425.12
Pig	SRC	7	121.23	1760.96
Pig	SRC	7	48.81	2430.01
Pig	SRC	7	0.70	41.61
Pig	HH	7	149.72	1546.48
Pig	HH	7	301.41	1744.13
Pig	HH	7	45.89	2058.93
Pig	HH	7	133.30	2050.81
Pig	HH	7	39.70	647.50
Pig	HH	7	59.97	494.22
Pig	HH	7	331.01	2126.93
Pig	HH	7	76.79	951.10
Pig	HH	7	155.34	2384.91
Pig	HH	7	210.99	1901.94
Pig	HT	7	103.18	1043.52
Pig	HT	7	32.34	344.31
Pig	HT	7	79.00	2875.48
Pig	HT	7	60.02	831.15
Pig	HT	7	53.09	1132.33
Pig	HT	7	44.66	1242.84
Pig	HT	7	28.19	955.34
Pig	HT	7	146.54	2287.75
Pig	HT	7	3.72	0.00
Pig	HT	7	118.57	2040.92
Pig	HH+HT	7	121.98	715.50
Pig	HH+HT	7	173.02	2314.48
Pig	HH+HT	7	27.80	544.88
Pig	HH+HT	7	298.86	1797.53
Pig	HH+HT	7	60.87	1011.86
Pig	HH+HT	7	14.36	824.04
Pig	HH+HT	7	228.39	1742.24
Pig	HH+HT	7	0.00	0.00
Pig	HH+HT	7	55.95	651.58
Pig	HH+HT	7	No Sample	
